# Nodular Inflammatory Foci Are Sites of T Cell Priming and Control of Murine Cytomegalovirus Infection in the Neonatal Lung

**DOI:** 10.1371/journal.ppat.1003828

**Published:** 2013-12-12

**Authors:** Felix R. Stahl, Katrin Heller, Stephan Halle, Kirsten A. Keyser, Andreas Busche, Anja Marquardt, Karen Wagner, Jasmin Boelter, Yvonne Bischoff, Elisabeth Kremmer, Ramon Arens, Martin Messerle, Reinhold Förster

**Affiliations:** 1 Institute of Immunology, Hannover Medical School, Hannover, Germany; 2 Institute of Virology, Hannover Medical School, Hannover, Germany; 3 Helmholtz Zentrum München, Institut für Molekulare Immunologie, München, Germany; 4 Department of Immunohematology and Blood Transfusion, Leiden University Medical Center, Leiden, The Netherlands; La Jolla Institute for Allergy and Immunology, United States of America

## Abstract

Neonates, including mice and humans, are highly susceptible to cytomegalovirus (CMV) infection. However, many aspects of neonatal CMV infections such as viral cell tropism, spatio-temporal distribution of the pathogen as well as genesis of antiviral immunity are unknown. With the use of reporter mutants of the murine cytomegalovirus (MCMV) we identified the lung as a primary target of mucosal infection in neonatal mice. Comparative analysis of neonatal and adult mice revealed a delayed control of virus replication in the neonatal lung mucosa explaining the pronounced systemic infection and disease in neonates. This phenomenon was supplemented by a delayed expansion of CD8^+^ T cell clones recognizing the viral protein M45 in neonates. We detected viral infection at the single-cell level and observed myeloid cells forming “nodular inflammatory foci” (NIF) in the neonatal lung. Co-localization of infected cells within NIFs was associated with their disruption and clearance of the infection. By 2-photon microscopy, we characterized how neonatal antigen-presenting cells (APC) interacted with T cells and induced mature adaptive immune responses within such NIFs. We thus define NIFs of the neonatal lung as niches for prolonged MCMV replication and T cell priming but also as sites of infection control.

## Author Summary

Neonates are highly susceptible to a number of infections that usually cause disease only in immunocompromised individuals, most likely because of their incompletely developed immune system. Although this phenomenon has been frequently observed, immune responses of neonates remain largely undefined upon infections with viruses. There is lack of knowledge about the spatio-temporal dynamics of host-virus interaction, especially in comparative infection models of neonates and adults. In this study, with the use of virus reporter mutants, we provide elaborate insight into these aspects in the mouse model of CMV infection. We define hallmarks of virus tropism, early cellular immune responses and general infection dynamics, findings that are fundamental to understand neonatal antiviral immunity. Furthermore, we found that neonatal APCs induce T cell responses in nodular inflammatory foci of the lung, a process which was supposed to be restricted to lymphoid organs. However, the MCMV-specific T cell response was qualitatively different in neonates from that in adults, possibly explaining - in part - the higher susceptibility of newborns. These observations expand our understanding of where adaptive immunity can be initiated, highlights the importance of early local cellular immune responses and sheds more light on neonatal antiviral immunity.

## Introduction

CMV infection shows an extraordinary high prevalence worldwide which increases with age [1], [2], but the majority of infected humans stays asymptomatic. Clinical symptoms dominate in neonates who suffered from congenital infection, postnatal infection of preterm low birth-weight infants, or in immuno-compromised adults [1], [3], [4], [5], [6]. The prevalence of CMV infection is already high in the very young who seem to be carriers of high viral loads and participate in the shedding of virus [7]. These observations imply that CMV infection is not sufficiently controlled by the immune system at the very early life. Accordingly, neonatal mice are more susceptible to infections with MCMV than adult mice [8], [9], [10], [11], [12], [13], [14]. Similar findings have been reported for other pathogens including Respiratory Syncitial Virus, Listeria monocytogenes, Herpes Simplex Virus type 1, Influenza Virus, and Pneumocystis [15], [16], [17], [18] suggesting that neonatal mice in general are more vulnerable to infections. The mechanisms behind this phenomenon as well as the differences in antiviral immunity between the very young and adults remain largely undefined [19], [20].

To understand and predict the outcome of a virus infection, it is of great importance to know where the infection is localized and what types of antiviral immune responses are initiated locally. Human cytomegalovirus (HCMV) DNA has been detected in several body fluids like blood, breast milk, saliva, urine, and bronchoalveolar fluid [3], [5], [7]. Thus, mucosal surfaces are most likely a primary target of postnatal CMV infection and indeed several routes of virus transmission have been suggested in neonates and children. Oral infection by contaminated breast milk and droplet infection of the lung by infectious saliva have been proposed in several studies [21], [22], [23], [24], [25]. MCMV has been widely used to investigate CMV infection *in vivo* in the mouse model [24], [26]. Infections have been extensively studied in adult mice after systemic administration of the pathogen while the natural infection routes of MCMV, including transmission to newborns, remains a matter of debate [27]. Therefore, it is still unknown which mucosal tissues are targets for viral entry and which cell types become infected to such a challenge.

Studying MCMV infection in adult mice has unmasked many aspects of the complex interplay between this pathogen and the immune system. Whereas CD8^+^ T cells are supposed to be major effectors of the host to control of MCMV infection there is also strong evidence that NK cells as well as CD4^+^ T cells contribute to keep the virus from undisturbed replication [24], [28]. However, the composition of the neonatal immune system seems to differ in many ways if compared to that of adults [29], [30]. This phenomenon is supplemented by the fact that there are enormous changes of immunity during the very first steps of life, especially in mucosal tissue [31]. However, the features of antiviral immunity in neonates infected with MCMV have not been investigated in detail. Accordingly, it is currently not known how the neonatal immune system responses to MCMV infection and why neonates subsequently suffer from increased morbidity and mortality.

Here, we investigated the primary virus tropism in mucosal surfaces of neonatal mice with the use of recombinant viruses expressing suitable reporter proteins [32]. Comparative analysis of lung infection in neonatal and adult mice reflected characteristics of HCMV infection in terms of virus tropism and histopathology. Accordingly, primary mucosal MCMV infection in neonatal mice led to a pronounced systemic viral spread and simultaneously caused disease, whereas adults rapidly coped with the infection. The clonal expansion of MCMV-specific CD8^+^ T cells in both adults and neonates was paralleled by virus control although the time course differed between the two groups. Interestingly, MCMV infection attracted myeloid cells to form morphological unique nodular inflammatory foci (NIF) in the neonatal lung. Within these structures MCMV-infected cells were destroyed and subsequently engulfed by local APCs. Notably, using *in situ* 2-photon microscopy, we visualized priming of naïve CD8^+^ T cells in NIFs of the neonatal lung suggesting that the neonatal organism allows the local differentiation of myeloid cells into APCs that directly cross-present antigen within NIFs. Thus, this study provides fundamental new insights in early antiviral immune responses during mucosal infections of neonatal mice.

## Results

### The neonatal intestinal mucosa is non-susceptible to MCMV infection

**Figure 1:**
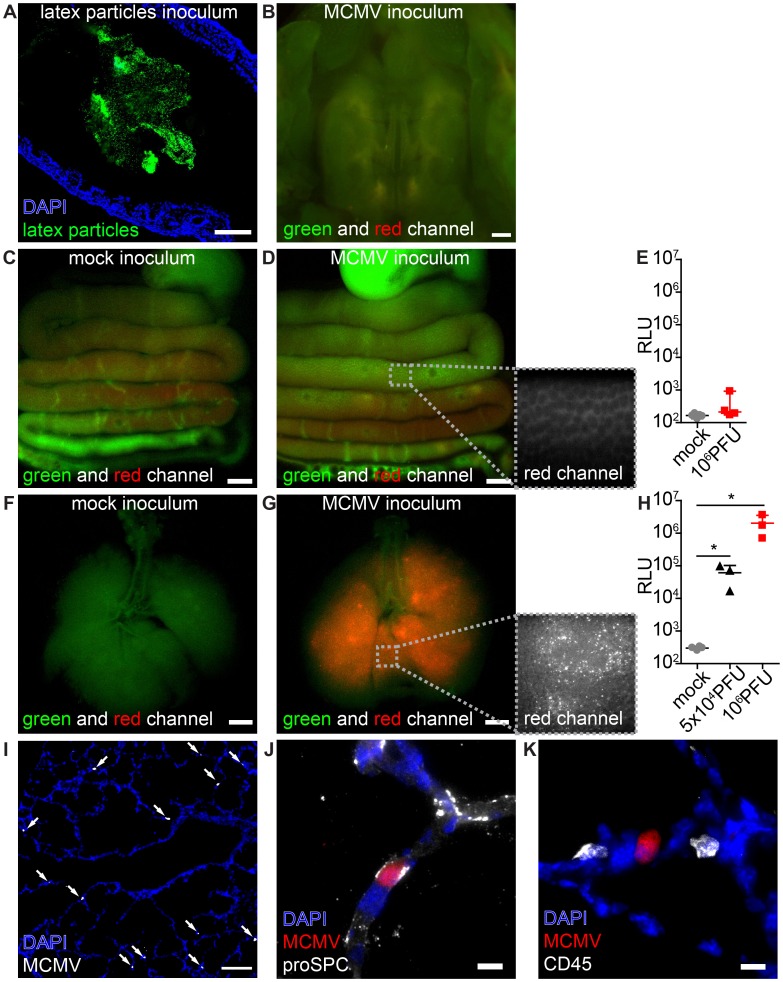
MCMV infects the neonatal lung but not the mucosa of the gastrointestinal tract. Neonatal mice were (A–E) fed or (F–K) l.p. infected with either (A) latex microspheres, (C, E, F, H) mock inoculums, (H, I–K) 5×10^4^ PFU or (B, D, E, G, H) 10^6^ PFU of MCMV-3D and analyzed one day after application. (A) Histological analysis of distal colon after inoculation of latex microspheres. (B) Oral cavity with bony palate was analyzed by epifluorescence microscopy, green and red channels show autofluorescent tissue. (C–E) Intestines were explanted from the proximal esophagus to distal colon in one piece, (F–H) respiratory tract with trachea. (C) Mock inoculum and (D) MCMV-3D inoculum show only autofluorescent tissue but no mCherry^+^ cells in the intestine, inlay in (D) shows autofluorescence in the red channel in high magnification. Luciferase activity from homogenates of (E) flushed intestines or (H) lungs. (F) Mice after mock inoculation show autofluorescent tissue or (G) multiple mCherry^+^ cells, inlay displays single mCherry^+^ cells in high magnification. (I–K) Frozen sections of infected neonatal lungs. (I) Overview with solitary mCherry^+^ infected cells (arrows). (J, K) Solitary mCherry^+^ infected cell with (J) pro-surfactant protein C (proSPC) or (K) CD45 staining. (A–D, F, G, I–K) Representative from >3 experiments with n = 2–3 animals per experiment. (E) Pooled from two independent experiments, n = 4–5, median & range. (H) Pooled from two independent experiments, n = 3–4, median & range. Scale bars: (B–D, F, G) 1000 µm, (A, I) 100 µm and (J, K) 10 µm.

To investigate which mucosal surfaces of neonatal mice can be infected we applied MCMV via different routes. Since virus transmission by contaminated breast milk has been reported in humans [22], [25], we firstly analyzed whether the mucosa of the gastrointestinal tract is susceptible to infection. Following oral application, fluorescent latex microspheres (0.5 µm in diameter, applied in PBS) were detected in the distal colon within 24 h, confirming sufficient ingestion of the inoculum (Figure 1A). When we fed neonates with high doses (10^6^ PFU) of the recombinant MCMV-3D that encodes the fluorescent protein mCherry as well as *Gaussia* luciferase [32] we could neither detect mCherry^+^ infected cells in the oral cavity (Figure 1B), esophagus, stomach, small or large bowel (Figure 1C+D) nor luciferase activity in organ homogenates of the gastrointestinal tract (Figure 1E). We therefore concluded that carrier-free MCMV does not infect the neonatal intestine via the oral route.

### Lung epithelial cells are highly susceptible to MCMV

Pneumonitis is a frequently observed manifestation of HCMV infection [5], [33], [34]. It has been previously reported that MCMV does infect the lung of adult mice after intranasal and subcutaneous application as well as the adult and neonatal lung after intraperitoneal (i.p.) injection [21], [35], [36], [37]. To investigate whether the lung mucosa could be a direct target for virus infection in neonatal mice we established a procedure to infect the lung by inoculation of virus into the laryngopharynx (designated as “laryngopharyngeal (l.p.) infection” throughout the article). High numbers of mCherry^+^ infected cells and dose-dependent expression of luciferase in organ homogenates were detected in the lung within 1 day after virus application (Figure 1F–H). Histological analysis showed solitary infected cells mainly in the epithelium of distal respiratory ducts and terminal bronchioles but not in the epithelium of the trachea (Figure 1I). Approximately 50% of the infected cells were pro-surfactant protein C-expressing type 2 alveolar epithelial cells [38] (Figure 1J, Figure S1A). No CD45^+^ cells of hematopoietic origin were found to be infected (Figure 1K, Figure S1B). Together, the data illustrate that the neonatal lung epithelium is highly susceptible to MCMV infection.

### Cytomegalovirus disease is associated with systemic infection in neonatal mice

**Figure 2:**
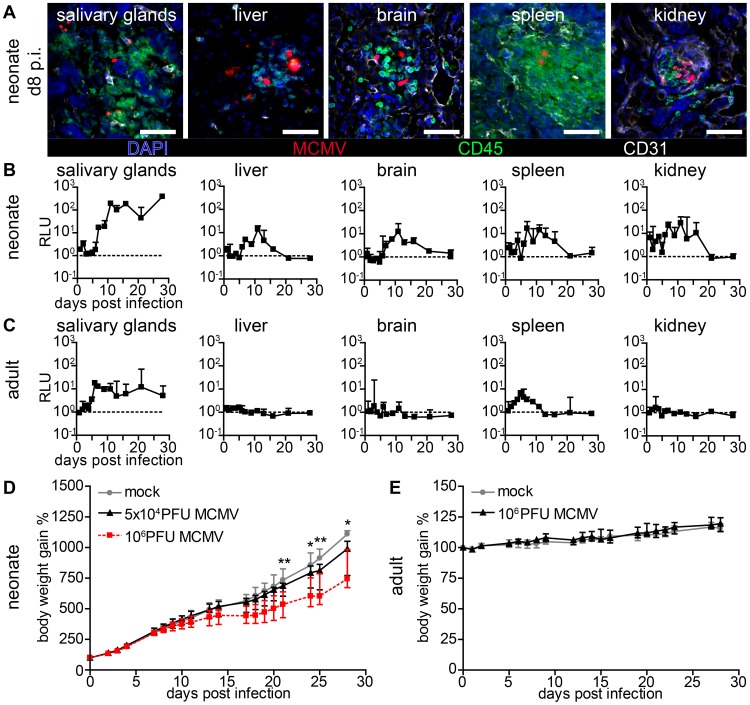
Viral dissemination establishes infection in multiple organs in neonatal but not in adult mice. (A–C) Neonatal or adult mice were infected as follows: neonates l.p. with 5×10^4^ PFU MCMV-3D, adults intranasal with 10^6^ PFU MCMV-3D. (A) Frozen sections of explanted neonatal organs were performed at day 8 after infection and analyzed after indicated antibody and DAPI staining, n>2 animals from >2 independent experiments. (B, C) Animals were sacrificed at indicated time-points after infection and homogenates of single organ preparations were measured for luciferase activity, shown are median & range and connecting line of medians, n = 3 animals per indicated time-points >3 independent experiments, dashed line = detection limit. (D+E) Body weight gain after MCMV-3D infection in (D) neonatal or (E) adult mice with the virus doses as indicated, median+IQR, pooled from two independent experiments each, n = 6–8 per group. Student's t-test between (D) mock and 5×10^4^ PFU group and (E) mock and 10^6^ PFU group.

Human CMV infection has not only been described in the lung but also in numerous organs and tissues such as the liver, brain, spleen, vascular endothelium and the kidney [3], [5], [39]. Likewise, in models for systemic MCMV infection multiple organs have been reported to become infected [10], [14], [35], [40], [41], [42]. We analyzed various organs after lung infection and found that by day 8 post infection (p.i.) mCherry^+^ cells could be detected in all investigated neonatal organs (Figure 2A). As described above for the lung, most infected cells were CD45^−^ demonstrating their non-hematopoietic origin. Various parenchymal cells were found to be infected and the spatial proximity to CD31^+^ vascular endothelial cells suggested hematogenous virus dissemination (Figure 2A). Surprisingly, we also found infected cells next to CD31^+^ vascular endothelial cells in the intestine (Figure S2A) indicating that hematogenous viral spread can lead to infection of the intestine, while oral application of the virus failed to infect the gut (see Figure 1). To gain insight into the dynamics of viral dissemination we determined luciferase activity to screen for virus spread to various organs following primary infection of the lung. All screened organs of neonatal mice including the salivary glands, liver, brain, spleen, and kidney possessed luciferase activity after primary lung infection (Figure 2B). However, whereas infection of the lung was already detectable one day p.i. (Figure 1H) viral activity in these organs was delayed by approximately six days (Figure 2B), an observation that also supports the idea of a barrier function of the lung preventing massive viral systemic exposure [43]. A comparative analysis with weight-adapted virus doses revealed that in adult mice luciferase was detected only in organ homogenates of the salivary glands and the spleen after intranasal infection with MCMV-3D (Figure 2C). Apart from the salivary glands, which have been described to be a place of ongoing virus persistence [44], [45], none of the tissues examined, neither neonatal nor adult, showed luciferase activity three weeks after infection (Figure 2B+C). Therefore, neonates as well as adults are able to cope with respiratory cytomegalovirus infection. However, when monitoring the body weight as a parameter of the health status, differences between adult and neonatal mice could be observed. While in the latter both a low dose (5×10^4^ PFU) as well as a high dose (10^6^ PFU) MCMV-3D infection temporally coincided with the diminished increase in body weight when compared to mock infected animals (Figure 2D), there was no effect in this respect in MCMV-3D-infected (10^6^ PFU) adult mice (Figure 2E). The pronounced vulnerability of neonatal mice to MCMV infection can be explained by productive virus infection in all organs that is accompanied by cachexia. We continued with low dose (5×10^4^ PFU) lung infections in further experiments in neonates to avoid excessive virus exposure to the neonatal organism.

### Formation of “nodular inflammatory foci” in the neonatal lung

**Figure 3:**
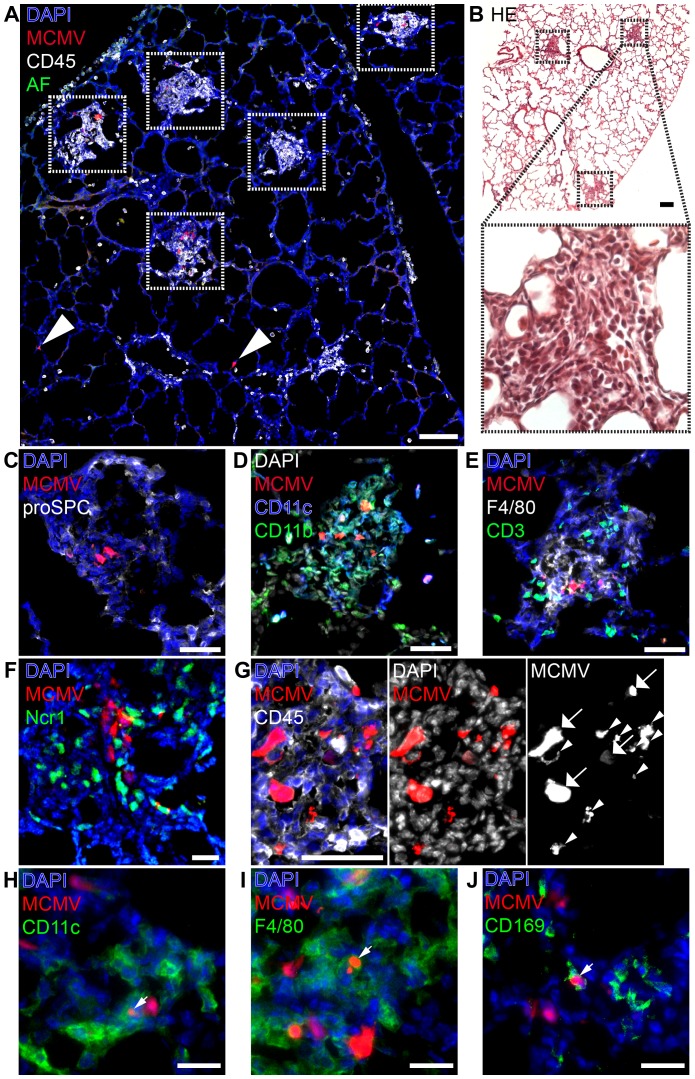
Characterization of “nodular inflammatory foci” in the neonatal lung 5 days after MCMV-3D infection. (A–E, G–J) Wildtype or (F) Ncr1^gfp/+^ neonatal mice were l.p. infected with 5×10^4^ PFU MCMV-3D, 5 days later mice were sacrificed and lungs explanted. (A, C–E, G–J) Frozen sections were stained with antibodies as indicated and DAPI nuclear staining. (B) Paraffin embedded sections were analyzed after hematoxylin and eosin staining. (C–E) Cellular composition of nodular inflammatory foci. (F) Frozen sections from Ncr1^gfp/+^ mice were analyzed by 2-photon microscopy. (A, B) Overview with multiple nodular inflammatory foci (frames in A and B) and solitary mCherry^+^ infected cells (arrowheads in A), (B) one nodular inflammatory focus is magnified. (G) Morphological discrimination between viable infected cells (arrows) and remnants (arrowheads) of infected cells. (H–J) Remnants engulfed by phagocytes are indicated by arrowheads. Scale bars: (A+B) 100 µm, (C–E, G) 50 µm, (F, H–J) 20 µm. AF = autofluorescence of tissue, (A–J) representative of n>6 from >3 independent experiments.

We speculated that an impaired antiviral immune response in the neonatal lung might be the cause of the massive systemic viral spread and disease. Therefore, we investigated early cellular immune responses in neonates via histological analysis of the lung 5 days after low dose infection (MCMV-3D; 5×10^4^ PFU), prior to the onset of viral dissemination. At this time-point numerous areas containing multiple infected cells with dense infiltration of CD45^+^ cells could be detected (Figure 3A, framed areas). These infiltrates were exclusively found around foci of infected cells, apparently sheathing them and causing a nodular appearance of the lung. Similar histopathology has been described in pulmonary HCMV-infection of immuno-compromised adults and termed “nodular inflammatory foci” [46]. Therefore, we equally termed these areas of the neonatal lung “nodular inflammatory foci” (NIF), defined as multiple juxtapositioned MCMV-infected cells and associated immune cell infiltrate.

Besides the presence of NIFs we found no evidence for further profound pathological alterations of the lung (Figure 3B). However, we frequently observed some solitary infected cells within the lung that were not contacted by CD45^+^ cells suggesting that they may escape immune surveillance (Figure 3A, arrow heads). Alternatively, these cells could also reflect earliest stages of NIF development. Most of the infected cells within the NIFs were neither pro-surfactant protein C-expressing type 2 alveolar epithelial cells nor CD45^+^ hematopoietic cells. Instead, cell morphology and position matched that of stromal cells such as fibroblasts (Figure 3C). Hence, in addition to type 2 alveolar epithelial cells also other cell types become infected during the course of infection.

Further analysis of the infiltrated cells identified them as a variety of CD11b^+^, CD11c^+^ and/or F4/80^+^ myeloid cells and the abundance of these cells account for the “nodular” morphology of the inflammatory foci (Figure 3D+E). In contrast, only few T and B cells were present (Figure 3E, Figure S3A–C). Natural killer cells were also present as illustrated by analysis of Ncr1^gfp/+^ transgenic mice (Figure 3F) and NK1.1 cell surface expression (Figure S3D). Further high resolution analysis revealed heterogeneous patterns of mCherry signals within the NIFs. The mCherry fluorescence intensity varied between infected cells suggesting that lung parenchymal cells were either differently permissive to infection or had been infected at different time-points and therefore were in different phases of viral replication (Figure 3G; arrows). In addition to multiple infected cells we found some cellular mCherry^+^ remnants that most likely were derived from infected apoptotic cells as described previously for MCMV-infected cells in the salivary glands [47] (Figure 3G; arrow heads). Interestingly, we found some of these remnants to be situated within CD45^+^ cells suggesting engulfment of mCherry-containing cell debris by myeloid cells (Figure 3G). We found remnants in CD11c^+^ cells and F4/80^+^ macrophages (Figure 3H+I). CD169^+^ macrophages were also present in NIFs at high frequencies and similarly contained mCherry^+^ remnants (Figure 3J). In contrast, few CD103^+^CD11c^+^ DCs were found to be present in NIFs but occasionally formed close contacts with infected cells (Figure S3E). Thus, NIFs are clearly different from bronchus-associated lymphoid tissue (BALT), tertiary lymphoid structures of the lung that are localized next to bronchi and characterized by the presence of B cell follicles with separated T cell areas [48], [49]. Instead, NIFs appear to be areas of viral replication although myeloid cells present in NIFs can engulf remnants of infected cells and probably function as APCs.

### Delayed MCMV control in nodular inflammatory foci of the neonatal lung

**Figure 4:**
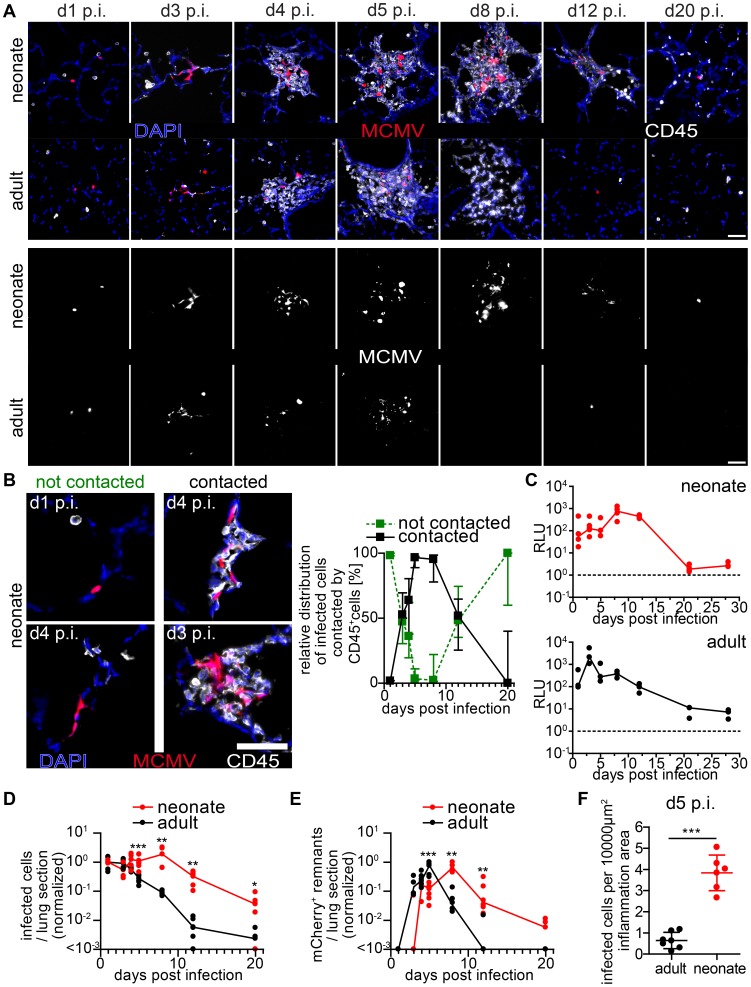
Delayed control of lung MCMV infection in neonatal mice. Neonatal (A–F) or adult (A, C–F) mice were infected as follows: neonates l.p. with 5×10^4^ PFU MCMV-3D, adults intranasal with 10^6^ PFU MCMV-3D. (A–F) Animals were sacrificed at indicated time-points after infection and frozen sections of explanted lungs were performed and analyzed after antibody and DAPI staining. (A) Upper rows show merged images of immune infiltrates associated with MCMV-infected cells (mCherry^+^), lower rows only mCherry signals. (B) Examples and relative distribution of infected cells that are either “contacted” or “not contacted” by neonatal immune cells. (C) Luciferase activity of homogenized lung explants from n = 3–5 animals from 1–2 experiments per time point. (D+E) Number of mCherry+ viable cells or remnants per lung slice, determined by 2 counted slices per animal, normalized to (D) mean of infected cells determined at day 1 p.i or (E) normalized to the maximal value of remnants per group; zero counts were set to <10^−3^ to allow logarithmic illustration, t-test between groups at indicated time-points, n = 2–4 animals per indicated time-point from >3 independent experiments. (F) Number of mCherry^+^ viable cells per lung slice at day 5 p.i., frozen sections of n = 6–7 animals from 2 independent experiments. (B) Median & range, (C–E) each value and a connecting line of medians, (F) mean & SD.

To gain comprehensive insight into the genesis of the NIFs and their role in viral clearance, we performed a comparative analysis of neonatal and adult lung sections at different time-points after infection with MCMV-3D. Over an observation period of three weeks we constantly found solitary infected cells in the neonatal lung that were not in contact with any hematopoietic cell (Figure 4A+B). However, multiple infected cells could be detected side by side 3 days p.i. suggesting cell-to-cell spread of MCMV *in vivo* (Figure 4A+B). NIFs could be detected in the neonatal lung from day 3 until at least day 8 p.i. but disappeared, to a large extent, by day 12 p.i. (Figure 4A+B). Accordingly, luciferase activity as well as the number of infected cells per lung slice did not decrease within the first 8 days p.i. (Figure 4C+D). Instead, NIFs appeared to be niches of ongoing virus replication possibly by recruiting susceptible fibroblasts [50]. The disappearance of the NIFs was associated with declining numbers of infected cells (Figure 4A,B+D). Interestingly, solitary infected cells that were not targeted by immune cell infiltrates were still present three weeks after infection (Figure 4A+B). In addition, appearance of NIFs coincided with the presence of mCherry^+^ cell remnants indicating immune cell-mediated destruction of infected cells within these structures (Figure 4B+E).

To compare these findings to those in the adult lung, we intranasally infected 6–8 week old mice with a weight-adapted dose of MCMV-3D. As observed for neonates, the virus also infected type 2 alveolar epithelial cells (unpublished data) and a comparable infection pattern to that observed in neonates was evident in adult mice within the first 3 days p.i. (Figure 4A). Likewise, a localized inflammation with immune cell infiltration was found around foci of infected cells. Although the morphology, composition and localization of these infiltrates may differ from the ones found in neonates we also named these structures “NIFs” of the adult lung. However, in contrast to the situation in neonates, the number of remnants of infected cells peaked already at day 5 p.i. in adult NIFs and only few morphologically intact mCherry^+^ infected cells could still be identified (Figure 4A, D–F). By day 8 p.i. luciferase activity had decreased from a peak at day 3 p.i. (Figure 4C). Accordingly, only residuals of cell infiltrates remained but few solitary infected cells were still detectable (Figure 4A+D). Luciferase activity as well as a comparative quantification of infected cells and mCherry^+^ remnants per lung slice showed a clear delay of virus control in the neonatal lung as compared to adults (Figure 4 C–F). These data demonstrate that neonatal mice can cope with most of the infected cells in the lung. However, although neonates induce a cellular immune response and form NIFs, they suffer from a persistent lung infection for up to three weeks. During the first 8 days infiltrating immune cells in the neonatal lung tolerate ongoing infection and fail to prevent spread of the infection to neighboring cells whereas adult mice start to contain viral replication in the lung already within the first 4 days.

### Expansion of MCMV-specific CD8^+^ T cells is crucial for control of virus infection

**Figure 5:**
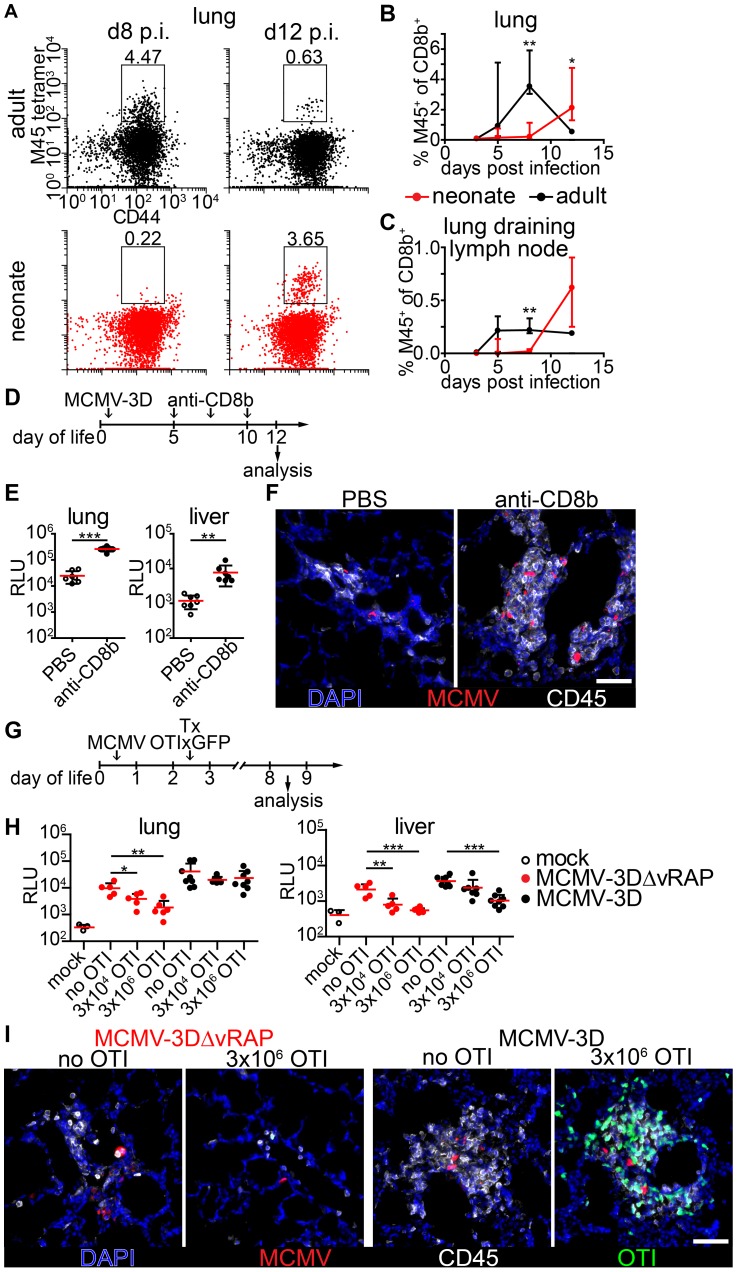
Delayed expansion of MCMV-specific T cells contributes to susceptibility of neonates. (A–C) Neonatal or adult mice were infected with either 5×10^4^ PFU or 10^6^ PFU MCMV-3D, respectively. Cell suspensions were generated from organs and time-points indicated and analyzed for the frequency of M45 tetramer-binding CD8^+^ T cell fraction. (A) Representative FACS plots and (B+C) quantification are depicted, median & IQR. (D) Experimental setup for E+F: neonatal mice were infected with 5×10^4^ PFU MCMV-3D, treated with anti-CD8b antibody or PBS and analyzed at the time-points indicated. (E) Luciferase activity of homogenates from organs depicted; means & SD. (F) Frozen sections of neonatal lung were analyzed with the antibodies indicated and representative images of NIFs are depicted. (G) Experimental setup for H+I: neonatal mice were infected with either 5×10^4^ PFU MCMV-3D or MCMV-3DΔvRAP; 2 days after infection none, 3×10^3^ or 3×10^6^ naïve OTIxGFP T cells were adoptively i.p. transferred and animals were analyzed 6 days after transfer. (H) Luciferase activity of homogenates from depicted organs, means & SD. (I) Frozen sections of neonatal lung were analyzed with antibodies indicated; representative images of NIFs or NIF residues. (A–C) Data from n = 3–5 animals per time-point from 2 independent experiments, (H+I) data from n = 5–8 animals from 5 independent experiments. (C) Scale bars, 50 µm.

CD8^+^ T cells have been implicated as major contributors to MCMV infection control in adult mice [28]. To test the hypothesis that the ongoing MCMV infection in the lung of neonates from day 1 until day 8 p.i. could be due to a limitation in the CD8^+^ T cell response we analyzed the presence of CD8^+^ T-cells which recognize the immunodominant MCMV epitope M45 [51]. M45-specific CD8^+^ T cells showed a massive expansion at day 8 p.i. in adult mice and already turned to the contraction phase at day 12 p.i. (Figure 5A–C). In contrast, M45-specific CD8^+^ T cells in neonates were hardly detectable before day 12 p.i. in lung or lung draining lymph nodes (Figure 5A–C). As the expansion of M45-specific CD8^+^ T cells showed a temporal coincidence with the initiation of virus control in neonates at ∼day 12 p.i. (Figure 4A–E) we depleted CD8^+^ T cells to investigate their importance in the clearance of infection (Figure 4D and Figure S4A). After depletion, we found higher luciferase activity in the lung, liver and all other organs analyzed (Figure 5E, Figure S4B). In addition, unlike the control group, CD8^+^ T cell-depleted neonatal mice showed NIFs which contained increased numbers of viable infected cells (Figure 5F) confirming the contribution of CD8^+^ T cells to controlling MCMV infection in neonatal mice.

Next, we performed adoptive transfers of CD8^+^ T cells into MCMV-infected neonatal mice to determine whether this treatment could abrogate MCMV infection. For this, we took advantage of lymphocytes from transgenic mice (OTI), which express a recombinant, K^b^-restricted T cell receptor that exhibits high affinity to MHC class I bound SIINFEKL peptide [52]. To that end, we infected neonatal mice either with MCMV-3D or MCMV-3DΔvRAP virus mutants [32]. Both viruses encode the SIINFEKL peptide but MCMV-3DΔvRAP lacks the “viral regulator of antigen presentation” genes encoding for the MHC class I evasion proteins gp48/m06 and gp40/m152 [53], [54]. In contrast to MCMV-3D, this mutant is therefore expected to lack the ability to interfere with MHC class I peptide surface expression as was recently shown for a related virus mutant [55]. We speculated that MHC class I bound SIINFEKL peptide presentation on MCMV-3DΔvRAP-infected cells would allow direct recognition of infected cells by OTI T cells and decreased luciferase activity in organs of these animals would be an *in vivo* indicator for the activity of cytotoxic T lymphocytes (CTL). Two days p.i. and at the day of infection we adoptively i.p. transferred various numbers of naïve CD8^+^ T cells from OTIxGFP mice and analyzed the animals six and ten days after transfer, respectively (Figure 5G and Figure S5A). In animals infected with MCMV-3DΔvRAP the reduction in luciferase activity in the lung and liver depended on the number of OTI T cells transferred (Figure 5H). Moreover, MCMV-3DΔvRAP-infected animals which received high numbers of OTI T cells showed no NIFs and only very few solitary infected cells could be found in the neonatal lung (Figure 5I and Figure S6C). The antiviral effect was also observed in the spleen, kidney, and brain of these mice (Figure S6B). In contrast, we did not observe a robust impact on luciferase activity within the neonatal lung and most organs tested 6 or 10 days after adoptively transferring OTI T cells into MCMV-3D-infected mice (Figure 5H, Figure S5A+B). Even in the presence of adoptively transferred OTI T cells these mice still possessed NIFs that harbored multiple infected cells (Figure 5I and Figure S6C). However, in MCMV-3D-infected neonatal animals, the liver significantly benefited from the transferred cytotoxic T lymphocytes (Figure 5H and Figure S5B). Previous reports have shown that already the deletion of one gene (m152) for the MHCI immune evasion leads to virus attenuation even in BALB/c neonatal mice [56]. In line with this report, in our model with infection of C57BL/6 neonatal mice we saw a trend to lower luciferase activity in the lungs of MCMV-3DΔvRAP-infected mice if compared to MCMV-3D-infected neonates, but the difference was not significant (Figure 5H, “MCMV-3D no OTI” vs. “MCMV-3DΔvRAP no OTI”, p = 0.0675, unpaired t-test). In summary, the CD8^+^ T cell response to MCMV infection in neonates is strikingly different from the response in adults and likely contributes to delayed virus control in neonates.

### Nodular inflammatory foci are sites of T cell priming

**Figure 6:**
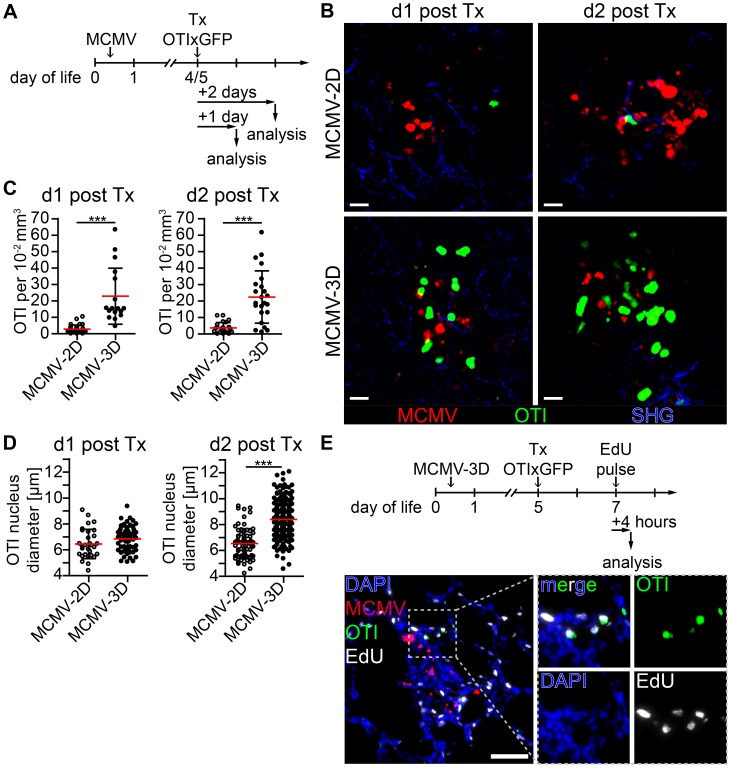
Early activation and proliferation of naïve OTI T cells in nodular inflammatory foci of the neonatal lung. (A) Experimental setup for B–D: neonatal mice were infected with either 5×10^4^ PFU control MCMV-2D or SIINFEKL-encoding MCMV-3D, 4–5 days after infection 5×10^6^ naïve OTIxGFP T cells were adoptively i.p. transferred and lungs of animals were analyzed 1–2 days after transfer. (B) Representative 2-photon microscopy images from Movie S1 and Movie S3 of either MCMV-2D or MCMV-3D-infected animals are depicted. (C) Quantification of OTIxGFP T cells per 2-photon microscopy 10^−2^ mm^3^ view field at indicated time-points, one dot represents number of OTIxGFP per view field at first time-point of Movie. (D) Maximal nuclei diameter of GFP^+^ cells (DAPI stain) within nodular inflammatory foci was measured in frozen histology sections, one dot represents one nucleus. (E) Experimental setup of T cell proliferation assay: neonatal mice were infected with 5×10^4^ PFU MCMV-3D. Five days after infection 5×10^6^ naïve OTIxGFP T cells were adoptively i.p. transferred, 2 days after transfer a subcutaneous pulse of EdU was applied and animals were sacrificed within four hours. Representative image of frozen sections of neonatal lung depicts EdU incorporation of OTIxGFP T cells within nodular inflammatory foci. (B–D) Data from n = 4–5 animals per group from 4 independent experiments. (E) Representative image of n = 2 animals from 2 independent experiments. (C, D) Means & SD. Scale bars (B) 20 µm and (E) 50 µm.

Priming of naïve CD8^+^ T cells is supposed to take place in secondary lymphoid tissue [57], and we have recently shown that T cells can also be primed in tertiary lymphoid tissues such as BALT [49]. Since we found many APCs in the NIFs of the neonatal lung, we wondered whether they could assist in priming naïve T cells already at the site of infection. To test this hypothesis, we adoptively i.p. transferred purified naïve CD8^+^ T cells from OTIxGFP mice into neonates infected with the reporter viruses MCMV-3D or MCMV-2D (Figure 6A). The latter lacks the sequence encoding the SIINFEKL peptide [32]. Within one day of transfer we found in lung draining lymph nodes of MCMV-3D, but not MCMV-2D-infected neonates, a considerable proportion of OTI T cells to express CD69, indicating T cell activation and arguing that neonatal lymph nodes are able to prime CD8^+^ T-cells (Figure S7A+B). To further investigate the priming capability of cells in the NIFs we performed *in situ* 2-photon microscopy of lung explants from MCMV-infected neonatal mice. Surprisingly, naïve OTI T cells accumulated already within 1 day after i.p. transfer in NIFs of MCMV-3D, but not of MCMV-2D-infected mice (Figure 6B+C, Movie S1). These T cells in MCMV-3D-infected neonates showed a slowed migration behavior in NIFs, similar to that of naïve T cells that are primed in the lymph node as reported earlier [58]. In contrast, peribronchial T cells which were not next to infected cells were not confined 1 day after transfer (Movie S2 - Scene 1). Within 2 days of transfer, a high proportion of OTI T cells in NIFs showed a lymphoblastic appearance and enlarged nuclei in MCMV-3D but not MCMV-2D-infected mice (Figure 6B–D, Movie S3). Additionally, after we subcutaneously treated MCMV-3D-infected neonates with a pulse of the nucleoside analog 5-ethynyl-2′-deoxyuridine (EdU) 2 days after T cell transfer and sacrificed the animals within 4 hours, immunohistology revealed a high frequency of proliferating EdU^+^ OTI T cells (Figure 6E). Furthermore, these T cells within NIFs became highly motile within 4 days of transfer (Movie S2 - Scene 2). Together, these data indicate that neonatal APCs in NIFs can induce OTI T cells to pass the classical priming program directly at the site of infection, including confined migration behavior after antigen-recognition, subsequent lymphoblastic appearance, cell proliferation and increased cell migration after the differentiation into CTLs.

### Neonatal APCs in NIFs can prime T cells independently from lymph nodes

**Figure 7:**
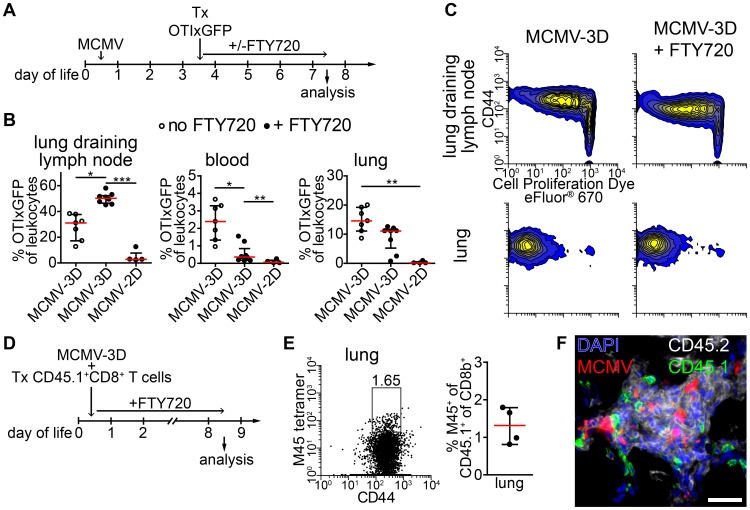
APCs in NIFs prime MCMV-specific CD8^+^ T cells. (A) Experimental setup for B+C: neonatal mice were infected with 5×10^4^ PFU MCMV-2D or MCMV-3D, 3 days p.i. 3×10^6^ naïve eFluor® 670 Proliferation Dye labeled CD8^+^ GFPxOTI T cells were adoptively i.p. transferred, some animals received daily s.c. FTY720 until analysis at day 7 p.i.. (B) Frequency of GFP^+^ cells in leukocytes in different compartments, mean & SD. (C) Representative proliferation profiles of GFP^+^ cells from organs indicated. (D) Experimental setup for (E+F): neonatal mice were infected with 5×10^4^ PFU MCMV-3D and at the same time received polyclonal CD8^+^ T cells from CD45.1^+^ mice. FTY720 was given daily subcutaneously. (E) Representative FACS plot and quantification of CD45.1^+^CD8^+^ T cells from lung stained with M45 tetramers, median & IQR. (F) Frozen sections of neonatal lungs were analyzed with antibodies indicated, representative image of a NIF. (B+C) Data from n = 4–7 animals from 2–3 independent experiments, (E–F) Data from n = 4 animals from 2 independent experiments. Scale bar: 50 µm.

Activated OTI T cells were detected as early as 48 hours after transfer within NIFs indicating that T cells were also primed in these structures. To formally exclude the possibility that activated T cells present in NIFs were initially primed in the lung-draining lymph node we blocked the egress of T cells from lymph nodes by treating neonates from the time of adoptive transfer of OTI T cells with the functional sphingosine 1-phosphate receptor antagonist FTY720 (Figure 7A) [59]. Four days after cell transfer, the frequency of OTI T cells in MCMV-2D-infected mice was extremely low in all compartments analyzed (Figure 7B). These cells did not proliferate and did not express the effector/memory marker CD44 (Figure S8A). In contrast, most of OTI T cells in lung draining lymph nodes of MCMV-3D-infected neonates had started to proliferate and expressed CD44, indicating that they experienced antigen (Figure 7C). Furthermore, we found significantly more OTI T cells in lung-draining lymph nodes of FTY720-treated MCMV-3D-infected neonates than in control animals and hardly detected OTI T cells in the blood of neonates which received FTY720, confirming the blockade of T cell egress from lymph nodes by this drug (Figure 7B). Despite the inhibition of T cell egress from lymph nodes, the frequency of OTI T cells in the lungs of FTY720-treated neonates was comparable to that in the control group (Figure 7B). Furthermore, OTI T cells in the lung of FTY720-treated animals showed proliferation and CD44 expression that were similar to those in the control group (Figure 7C). Conclusively, these data confirm the hypothesis that activated T cells present in NIFs have also been activated in these structures.

As these data suggested that neonatal APCs can potently prime T cells in lymph nodes and in NIFs we wondered if a small T cell receptor repertoire in neonates and therefore a low precursor frequency for MCMV-specific T cells accounted for the delay in clonal expansion of M45-specific CD8^+^ T cells (Figure 5A). Consequently, we adoptively transferred 10^7^ polyclonal CD45.1^+^CD8^+^ T cells from adults into neonates at the time of MCMV-3D infection and treated these animals with FTY720 to prevent egress of lymph node-primed T cells (Figure 7D). We speculated that the adult T cell repertoire contains T cells with M45-reactive TCRs and that this adoptive transfer would substitute for the missing MCMV-reactive CD8^+^ T cells in neonates. As clonal expansion of M45-specific T cells peaked at ∼8 days p.i. in adults (Figure 5A–C) we also analyzed neonates at day 8 p.i. (Figure 7D). Of interest, we found a considerable frequency of M45-specific T cells in the neonatal lung within the CD45.1^+^CD8^+^ T cell fraction (Figure 7E). In addition, the transferred CD45.1^+^ cells (with ∼90% of CD45.1^+^ cells being CD8^+^ T cells; unpublished data) were situated within NIFs suggesting the accumulation of MCMV-specific CTLs in NIFs (Figure 7F). In summary, these data support the hypothesis that APCs in neonatal NIFs are capable of presenting MCMV peptides (including M45) to naïve CD8^+^ T cells to directly prime these cells at the site of infection. Furthermore, as the adoptive transfer of polyclonal adult CD8^+^ T cells led to expansion of M45-specific clones, it is likely that the low precursor frequency of MCMV peptide-specific CD8^+^ T cells accounts for the delayed clonal expansion of M45-specific CTLs in neonates.

### Visualization of APC and T cell interaction in nodular inflammatory foci of neonatal mice

**Figure 8:**
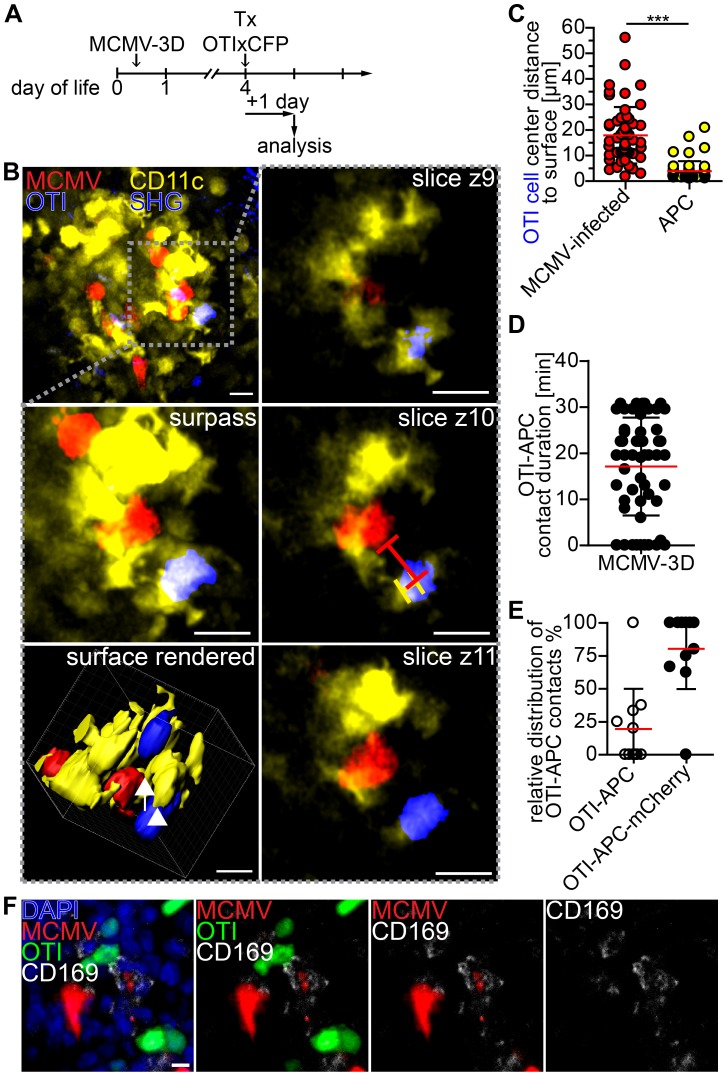
Visualization of cross-presentation within nodular inflammatory foci of neonatal mice. (A) Experimental setup: neonatal CD11c-YFP transgenic mice were infected with 5×10^4^ PFU SIINFEKL-encoding MCMV-3D, 4 days after infection 5×10^6^ naïve OTIxCFP T cells were adoptively i.p. transferred, 1 day after transfer lungs were analyzed by 2-photon microscopy. (B) Representative 2-photon microscopy image from Movie S4 of nodular inflammatory foci in surpass mode and z-axis sequential images from one time-point of framed area are depicted, lines in z4 indicate distances from OTI to APC or infected cell, arrow indicates synapse between infected cell (red) and APC (yellow), arrow head indicates synapse between OTI T cell (blue) and APC (yellow). (C) Distances from OTIxCFP T cell center to surface of either mCherry^+^ or APCs at first time-point of Movies are depicted. (D) Contact-duration of OTIxCFP with APCs was estimated from 12–31 min Movies. (E) Percentage of OTIxCFP T cells with APC contacts where APCs are either *in* or *not in* contact with mCherry^+^ signal. (F) Neonatal mice were l.p. infected with 5×10^4^ PFU MCMV-3D, 5 days p.i. 5×10^6^ naïve OTIxGFP T cells were adoptively i.p. transferred and one day later mice were sacrificed and lungs explanted, frozen sections were stained with indicated antibodies and DAPI. (C–E) Means & SD. (B–E) Data from n = 4 animals from 2 independent experiments, (F) representative from n = 4 animals from 3 independent experiments. Scale bars (B) 15 µm, (F) 10 µm.

Finally, we aimed to further characterize T cell priming in non-lymphoid tissue by 2-photon microscopy of NIFs in the neonatal lung. In particular, we wondered if we could observe interactions between APCs and CD8^+^ T cells. Since CD11c is mainly expressed by dendritic cells and alveolar macrophages [60], we infected neonatal CD11c-YFP transgenic mice with MCMV-3D. Four days later we adoptively transferred purified naïve CD8^+^ T cells from OTIxCFP mice (Figure 8A). Numerous OTI T cells could be found in a dense network of CD11c^+^ APCs in the NIFs within 1 day of T cell transfer (Figure 8B, Movie S4). Interestingly, OTI T cells were in direct contact with APCs, but only occasionally with cells infected with the reporter virus MCMV-3D that carries the MHC class I immune evasion genes and is therefore expected to interfere with MHC class I peptide presentation (Figure 8B+C). Most of the contacts observed between OTI T cells and APCs were stable and some lasted for more than 30 minutes (Figure 8D). Of interest, APCs formed cell protrusions which connected OTI T cells with infected cells (Figure 8B, Movie S4). Most of the contacts between APCs and OTI T cells occurred when the APC itself was in contact with an infected cell (Figure 8E). Histological analysis of NIFs revealed intensive synapse formation of OTI T cells with CD169^+^ macrophages which contained remnants of infected cells (Figure 8F). These data support the idea that MCMV-specific cytotoxic CD8^+^ T cells can be primed by myeloid cells, potentially by CD169^+^ macrophages, of virus-induced NIFs in the lung and that these myeloid cells contribute to the local antiviral immune response.

## Discussion

In the present study we established an animal model for mucosal MCMV infection in neonatal and adult mice and describe the spatio-temporal distribution of virus infection at the single-cell level. Compared to classical MCMV infection models using systemic application (i.p. or i.v.) of the virus that leads to primary infection of multiple organs, the approach used in this study allows investigation of immune responses at mucosal surfaces. Our data suggest that infection of the respiratory tract serves as a previously underestimated entry organ for CMV in neonates and that other organs become infected after primary virus replication and hematogenous spread. This virus is known to persist in salivary glands and infectious virus can be found in saliva of infected humans. Thus, CMV may be transmitted via virus-containing saliva to the respiratory tract. This transmission route may be of clinical importance especially for postnatal infection of highly susceptible preterm low birth-weight infants. In neonatal and adult lungs type 2 alveolar epithelial cells were frequently found to be infected with MCMV, a cell type that also has been suggested as a target for HCMV [61], [62]. Our observations are also in accordance with the model of cell-to-cell spread of infectious virions in the lung since we could visualize that different neighboring cell types became infected at later time-points after primary infection. The proximity of alveolar epithelial cells, fibroblasts and vascular endothelial cells within the lung [63] suggests the sequential infection of these cells as an imaginable route for virus particles to enter the blood stream from the pulmonary alveoli and spread systemically within the host. Indeed, infection of the gastrointestinal tract was evident in neonatal mice after hematogenous virus spread but not after virus administration via the oral route. After oral application, low pH in the stomach and bile in the duodenum supposedly prevent infection of the small and large intestine with the enveloped MCMV. Though, the neonatal stomach is not very acidic in the first days of life and therefore may allow the virus to enter the duodenum as an infectious particle. However, it was unexpected that neither the oral cavity nor the esophagus seem to be mucosal entry sites for MCMV. A study by Wu and colleagues suggested that neonatal mice can be infected after oral application of carrier-free MCMV as well as virus-containing milk cells [64]. However, these authors did not analyze infection of the gastrointestinal tract itself but instead found viral transcripts in the lung and other organs 4 days after oral delivery of virus. Therefore, it is currently not known which cells are first targeted by MCMV after oral application. Nevertheless, the data presented in our study suggest that after systemic spread from a primary site of infection such as the respiratory tract, MCMV can disseminate to any vascularized corner of the body and virus infection of the colon may actually occur from the “blood-side” and hike through the epithelium rather than start at the apical side of the epithelium.

To prevent, or at least reduce, systemic dissemination of pathogens efficient antiviral defense mechanisms has to be induced very early after infection of mucosal tissues. Following MCMV lung infection neonatal mice failed to prevent the systemic spread of virus originating from infected cells of the lung. An impaired first line antiviral defense is most likely the cause for the high susceptibility of neonates to virus infection and explains prolonged viral replication in the lung and pronounced virus dissemination with subsequent infection of various organs. Still, virus spread to other organs was usually not observed during the first 5 days of infection in neonates and in adults, and the majority of infected cells was cleared after 3 weeks suggesting that local immune responses of the neonatal lung to some degree help to reduce systemic spread of the virus.

Several of our observations support the hypothesis that the formation of NIFs contribute to control MCMV infection of the neonatal lung: i) infected cells of the lung were efficiently removed at locations where NIFs were induced, ii) remnants of infected cells were always found to be associated with NIFs, iii) macrophages within the NIFs contained fragments of lysed, virus-infected cells indicating that NIF macrophages locally remove infectious virions, and iv) NIFs provided an environment that allowed priming of antigen-specific cytotoxic T lymphocytes.

Immunohistology identified NIFs to primarily consist of MCMV-infected cells and myeloid cells including macrophages (F4/80^+^; CD169^+^) as well as DCs (CD11b^+^CD11c^+^; CD103^+^) while only few lymphocytes and NK cells were present. These features clearly distinguish NIFs from induced BALT that develops after the clearance of infections and is characterized by large and separated T and B cell zones and that has been shown to act as a general priming site for T cells [49]. Although T cells are sparse, our data indicate that APCs can efficiently prime naïve CD8^+^ T cells directly in NIFs. T cell priming is a multistep process which has been extensively characterized in secondary lymphoid organs such as lymph nodes. There, following recognition of antigen presented by APCs T cells undergo an extensive proliferation and differentiation program that lasts for at least three days. During this period all lymphocytes are trapped within these organs by a process known as lymph node shut down, which also prevents the release of activated T cells. Therefore, it is unlikely that those OTI T cells that were observed to rapidly proliferate in NIFs 2 days after their adoptive transfer were initially primed in lung-draining lymph nodes. The idea that T cells are directly primed in NIFs is further supported by the finding that proliferating T cells were also present in NIFs of FTY720-treated mice, where T cell egress from lymph nodes is blocked. Furthermore, 2-photon microscopy studies revealed intensive interaction and synapse formation of APCs and OTI T cells within NIFs. In addition, approximately 75% of the APCs that contacted OTI T cells simultaneously interacted with infected cells indicating that APCs, which are not actively infected, actually cross-present viral antigens to naïve T cells that differentiate to mature CTLs.

It is unknown which entry portal is used by naïve T cells to enter NIFs and what signaling molecules are involved. As data on essential molecules for homing of lymphocytes into the lung is sparse this needs to be addressed in future studies. Furthermore, it is currently unclear which subset of the CD11c^+^ APCs observed actually cross-presents antigen in the NIFs. We recently identified lung-derived CD103^+^ cells to cross-present antigen to CD8^+^ T cells in lung-draining lymph nodes [65]. Thus it seems possible that CD103^+^ DCs also cross present antigen directly in NIFs and indeed immunohistology identified few CD11c^+^ CD103^+^ DCs to be in direct contact with infected cells. Alternatively, some of the newly recruited monocytes and/or DC progenitors undergo a differentiation program within NIFs that allows the local generation of cross-presenting DCs. Interestingly, CD169^+^ macrophages were present at high frequencies within NIFs. CD169^+^ lymph node macrophages have recently gained considerable attention since they were identified to play important roles in controlling spread of lymph-derived virus, in presenting lymph-derived antigen to B cells and to cross-present lymph-derived apoptotic tumor cells to induce cytotoxic T cell responses [66], [67], [68]. In NIFs, CD169^+^ macrophages not only contained remnants but also contacted infected cells and simultaneously formed synapses with OTI T cells suggesting that these cells actually cross-present antigen and therefore contribute to the control of MCMV infection in the neonatal lung.

Antibody depletion of CD8^+^ T cells clearly promoted virus replication in MCMV-3D-infected neonates emphasizing an important role of cytotoxic T cells in MCMV control. As we observed activated endogenous CD8^+^ T cells in MCMV-infected neonates at day 8 (Figure 5A, CD44 expression and unpublished data) it is likely that MCMV epitopes were recognized by CD8^+^ T cells at that time. Additionally, the cytotoxic CD8^+^ T cells activated in NIFs are seemingly fully functional since naïve OTI T cells adoptively transferred into neonates differentiated to cytotoxic effector T cells and efficiently reduced the viral load in all organs analyzed of mice infected with MCMV-3DΔvRAP. Activated OTI T cells had only a limited effect in mice infected with the MCMV-3D variant. These data indicate that the MCMV-encoded vRAP proteins, m06 and m152, efficiently prevent killing of MCMV-infected cells by CD8^+^ T cells as shown by others before [56], [69], [70], [71], [72]. These observations suggest that, in principle, neonatal mice can prime CD8^+^ T cells and induce CTL-mediated antiviral immunity. Interestingly, the generation of CTLs in the present animal model is in line with a previous report showing expansion of CMV-specific CD8^+^ T cells in newborns upon HCMV infection [73]. This raises the question why particularly the CTL response should be responsible for the higher susceptibility of neonates to MCMV infection. First, the frequencies of lymphocytes are in general lower in neonatal than in adult mice [30]. Furthermore, our observation that M45-specific CD8^+^ T cells efficiently expand in neonates once adoptively transferred from adult donors suggest that a low precursor frequency - rather than a general defect in T cell priming in neonates - contributes to the late expansion of M45-specific CD8^+^ T cells. These findings are in line with reports showing that the neonatal TCR repertoire and hierarchy differ from that of adults [74], [75]. Therefore, low numbers of MCMV-specific CTLs in combination with a reduced clonal repertoire and diminished variety of recognized viral proteins may account for the vulnerability of these young organisms.

We also observed NK cells and CD4^+^ T cells in NIFs but it is currently unclear to what degree these cells contribute to the anti-MCMV response in these structures. NK cells have been proposed to lack multiple activating receptors during the very first days of life [76]. Additionally, neonatal myeloid cells have been reported to produce only low levels of IL-12 [77] and subsequent low IFN-γ responses by T cells and NK cells may diminish antiviral immunity in neonates. As CD4^+^ T cells also contribute to control of MCMV infection it is likely that low precursor frequencies of both, MCMV-specific CD4^+^ and CD8^+^ T cells, account for the vulnerability of neonates to MCMV infection. In summary, the high susceptibility of neonatal mice to viral infection may be the result of an impaired innate and a delayed adaptive antiviral immune response that allows prolonged local virus replication and extreme systemic viral spread with multi-organ disease and cachexia.

In both, the adult and neonatal lung solitary infected cells were still present when all the infected cells had been removed from the NIFs. These findings suggest the existence of micro-anatomical niches which allow immune evasion of infected cells. Likely, innate immune responses are needed to allow migration of immune cells to places of viral infection which then leads to removal of MCMV-infected cells or inhibition of viral replication. Possibly, in some cells MCMV infection does not trigger these early responses and therefore the first steps of inflammation are not initiated. Alternatively, solitary cells might result from secondary infections with viruses released from other organs such as the salivary glands. The fate of these infected cells, apparently ignored by the immune system, clearly deserves further attention since it cannot be excluded that latent infection is finally established in such cells.

The present study is to our knowledge the first to describe and profoundly characterize NIFs as well as solitary infected cells in the neonatal lung upon MCMV infection. Interestingly, CMV-associated interstitial pneumonia with formation of nodules is one among various reported lung manifestations of CMV infection in immuno-compromised adults [33], [46]. As it is currently unclear what factors determine the type of lung manifestations in human CMV patients, the mouse model presented in this study might help to shed light on the pathogenesis of CMV lung disease as well as the definition of crucial antiviral immune responses to control CMV infection in the lung.

In summary, this study provides profound insight into host-pathogen interaction upon viral challenge of the lung of neonatal mice. The localized accumulation of primarily myeloid immune cells at the site of infection represents an essential feature for the formation of NIFs in the neonatal lung. These structures allow the local induction of adaptive immune responses and moreover represent the anatomical correlate where the control of MCMV infection takes place.

## Materials and Methods

### Animals

Mice were all on a C57BL/6 background, bred at the central animal facility of Hannover Medical School under specific pathogen free conditions and/or purchased from Charles River Laboratories. ß-actin-eGFP mice [78] and ß-actin-eCFP [79] mice were crossed to ovalbumin-transgenic TCR (OTI) mice [52] and the F1 cross was labeled as OTIxGFP and OTIxCFP, respectively; CD11c-YFP [60]; Ncr1^+/gfp^ [80].

### Ethics statement

All animal experiments were performed according to the recommendations and guidelines of the Federation of European Laboratory Animal Science Associations (FELASA) and Society of Laboratory Animals (GV-SOLAS) and approved by the institutional review board and the Niedersächsische Landesamt für Verbraucherschutz und Lebensmittelsicherheit (AZ33.9-42502-04-10/0225 and AZ33.12-42502-04-12/0921).

### Viruses and infections

MCMV mutants have been described previously [32] and were produced and titrated on mouse embryonic fibroblasts. MCMV-2D encodes *Gaussia* luciferase and mCherry, MCMV-3D carries additionally a sequence within the m164 ORF encoding the SIINFEKL peptide. The MCMV-3DΔvRAP mutant is identical to MCMV-3D except that it lacks the m06 and m152 ORFs. All reporter viruses lack the m157 ORF that encodes a ligand for the activating receptor Ly49H present on NK cells in C57BL/6 mice [81]. C57BL/6 wildtype or CD11c-YFP mice were mated and dams were kept with their litter. Neonatal mice were infected on their first day of life (<24 h old); “oral” inoculations were performed by repeated moistening of the mouth with fluid up to a volume of 10 µl (for control applications we used 3×10^9^ Fluoresbrite YG Microspheres, Polysciences Europe GmbH), for l.p. inoculations a volume of 10 µl was administered by probing of the laryngopharynx with a pipette and extension of the neck. Adult C57BL/6 wildtype mice (6–8 weeks old) were anesthesized (100 mg/kg BW ketamine and 5 mg/kg BW xylazine) and 20 µl of virus solution was applied to each nostril for “intranasal” infection.

### T cell transfers, FTY720 and antibody treatment

CD8^+^ T cells were isolated with MACS CD8^+^ T cell isolation kit (Miltenyi Biotec) from lymph nodes and spleen of OTIxGFP or OTIxCFP mice and had a purity of 85–95%. MCMV-2D and MCMV-3D-infected neonatal mice received equal numbers (5×10^6^ cells) of naïve CD8^+^ T cells via i.p. application (Figure 6). CD8 T cells were depleted by intraperitoneal application of RmCD8.2 mAb (25 µg/g body weight; Figure S4A). FTY720 was given subcutaneously (5 µg/g body weight) on a daily basis. The first administration was given at the time of cell transfer (Figure 7).

### Leukocyte isolation from lungs

Right heart ventricle was perfused with PBS until blood cells were removed from the lung. Fragmented tissue was digested with Collagenase D (Roche, 0.5 mg/ml) and DNAse I (Roche, 0.025 mg/ml) for 45 min at 37°C, meshed through 40 µm Falcon® Cell Strainer and leukocytes isolated with Lympholyte®-M.

### Epifluorescence microscopy, histology and flow cytometry

Leica MZ16 epifluorescence microscope was used for whole organ images. For histology organs were fixed in 2% PFA and 30% sucrose for 30 min and embedded in OCT compound (Tissue-Tek, Sakura). 7 µm-thick organ slices were stained after appropriate blocking with depicted antibodies. Images were taken with an AxioCam MRm camera (Carl Zeiss) attached to Axiovert 200M fluorescence microscope (Carl Zeiss) with PlanApochromat objectives 10×/0,45, 20×/0,75 and 40×/0,95 (magnification/numerical aperture) and processed with AxioVision 4.8 software. Images of HE stained sections were taken with Olympus BX61 microscope and ColorView IIIu camera with UPlanSApo objectives (4×/0,16 and 40×/0,90) and processed with cell∧P 5.0 (Olympus Europe). All images were processed with Microsoft Office Picture Manager. Cell strainers (BD Falcon) were used to prepare suspensions for FACS analysis or cell purification from lymph node or spleen cells. Cells were processed with LSRII Cytometer and data was analyzed with BD FACSDiva Software (6.1.3) or WinList 6.0 software. The following antibodies (clones) were used after adequate blocking of Fc receptors: B220-Cy5 (RA3-3A1), CD103-PE (M290), CD169-AlexaFluor647 (MOMA-1), CD11b-AlexaFluor488 (M1/70), CD11c-APC (N418), CD3-AlexaFluor488 (17A2), CD3-PE (17A2), CD31-biotinylated (MEC13), CD4-biotinylated (GK1.5), CD4-PerCP (RM4-5), CD44-eFluor450 (IM7), CD45-APC (30-F11), CD69-PerCP/Cy5.5 (H1.2F3), CD8a-APC/Cy7 (53-6.7), CD8b-Cy5 (Rm CD8-2), CD8b-AlexaFluor488 (Rm-CD8-2), F4/80-APC (BM8), NK1.1-PE (PK136), pro surfactant protein C (AB3786) combined with anti-rabbit-Cy5 (Jackson ImmunoResearch), TCR-Vα2-PE (B20.1). Streptavidin-Cy5 (eBioscience), Streptavidin-APC/Cy7 (BD-Pharmingen), Cell Proliferation Dye eFluor® 670 (eBioscience), M45-tetramer-PE provided by Ramon Arens.

### Luciferase measurements

Single organ preparations were performed after perfusion of supplying blood vessels with PBS. Organs were kept in PBS, homogenized with TissueLyser II (Qiagen) and supernatants were measured for luciferase expression after addition of “native Coelenterazine” (Synchem) with Lumat LB 9507 (Berthold Technologies). For lung, salivary glands, gut and liver 1∶10 dilutions were performed for measurements. The following organs were analyzed: lung (Figure 1 complete lung, Figure 5 lobes of right lung including trachea), gut (from proximal esophagus to distal colon), salivary glands (all sublingual and submaxillary), brain (down to the bulb), spleen, liver (complete liver of neonates, only left lobe from adults), kidney (right only). Luciferase measurements of organs from non-infected animals were used as controls and data was normalized to means of control measurements to determine the detection limits.

### 2-photon microscopy

Neonatal lungs were explanted and 400 µm-thick lung slices were prepared with use of a Tissue Chopper (McIllwain). Lung slices were fixed on a imaging chamber using tissue adhesive (Surgibond) and kept in oxygenated (95% O_2_/5% CO_2_) RPMI 37°C medium (Invitrogen) containing 5 g/L glucose. Imaging was performed with Olympus BX51 upright microscope equipped with a 20×/0.95 water immersion objective. A MaiTai Ti∶Sa pulsed IR laser (Spectra-Physics) was set to 920 nm for excitation of eGFP (as well as Ncr1^gfp/+^ and DAPI for Figure 3 F) or 860 nm for excitation of eCFP and YFP. A second laser excited mCherry with 1100 nm generated from an optical parametric oscillator (OPO; APE, Berlin). Z-stacks of up to 30 images from 300×300×60–160 µm (Movie S1, S2, S3) or 150×150×60–160 µm (Movie S4) viewfields were acquired every 20–30 seconds to generate time-lapse series. Data was analyzed with Imaris 7.x (Bitplane Scientific Software) and processed with MAGIX Video deluxe 2013.

### Quantification of infected cells

7 µm-thick lung sections of 2–4 animals per time-point and group were performed at comparable anatomical positions (central lung, slices including right and left lobes and main bronchi). “Viable” infected cells were distinguished from cell “remnants” by the following criteria: morphology (smooth edge, round shaped with or without elongations), nucleus (clear non-fragmented DAPI signal present) and cell size (larger than 5 µm) (Figure 3F). Mean of two counted slices per animal was calculated for Figure 4D and E. Area of inflammation was determined by manual measurement of CD45^+^ stain signal using AxioVision 4.8 software (Figure 4F).

### EdU incorporation

Neonatal mice were subcutaneously injected with 125 µM EdU and sacrificed within 4 h after injection. Histological staining was performed with Click-iT EdU Imaging Kit (Invitrogen).

### Statistical analysis

Statistical analysis was performed with Prism 4 (Graph-Pad Software, Inc.). Unpaired t-test for comparison of 2 groups or ANOVA one-way analysis for >2 groups. Statistical significance was depicted as follows: *, p<0.05; **, p<0.01; and ***, p<0.001.

## Supporting Information

Figure S1 **MCMV cell tropism in the neonatal lung at 1 day post infection.** (A+B) Neonatal mice were l.p. infected with 5×10^4^ PFU MCMV-3D. One day later mice were sacrificed and lungs explanted. Frozen sections were stained with antibodies and DAPI as indicated. AF, autofluorescence of tissue. Representative from >3 experiments with n = 2–3 animals per experiment. Scale bars: 100 µm
(TIF)

Figure S2 **MCMV infection of the neonatal colon.** Neonatal mice were l.p. infected with 5×10^4^ PFU MCMV-3D. Intestines were explanted at day 8 after infection. Frozen sections were stained with antibodies and DAPI as indicated. Representative of n = 5 animals from 2 independent experiments. Scale bar: 50 µm.
(TIF)

Figure S3 **MCMV cell tropism in the neonatal lung at 5 days post infection.** (A–E) Neonatal mice were l.p. infected with 5×10^4^ PFU MCMV-3D. 5 days later mice were sacrificed, lungs explanted and frozen sections were stained with antibodies and DAPI as indicated. Arrows point to CD45^+^NK1.1^+^ NK cells (D) and CD11c^+^CD103^+^ dendritic cell (E). Scale bars: (A–D) 50 µm, (F) 20 µm.
(TIF)

Figure S4 **Depletion of CD8^+^ T cells impairs control of MCMV in neonates.** Neonatal mice were l.p. infected with 5×10^4^ PFU MCMV-3D and treated with anti-CD8b antibody or PBS. At day 12 p.i. animals were sacrificed. (A) Lung draining lymph nodes were analysed for the presence of CD8a^+^ T cells. (B) Luciferase activity was measured from homogenized organs as indicated; mean & SD. Data from n = 7 animals per group from 2 independent experiments.
(TIF)

Figure S5 **Viral activity 10 days after adoptive transfer of OTI T cell into neonates.** (A) Experimental setup for (B): Neonatal mice were l.p. infected with 5×10^4^ PFU MCMV-3D, received indicated numbers of CD8^+^ OTIxGFP cells intraperitoneally at the same day and were analysed at day 10 p.i. (B) Luciferase activity of homogenized organs as indicated, mean & SD, n = 4–5 animals per group from 2 independent experiments.
(TIF)

Figure S6 **Viral activity 6 days after adoptive transfer of OTI T cell into neonates.** (A) Experimental setup for (B+C): Neonatal mice were l.p. infected with 5×10^4^ PFU MCMV-3DΔvRAP or MCMV-3D, received indicated numbers of CD8^+^ OTIxGFP cells intraperitoneally at day 2 and were analysed at day 8 p.i. (B) Luciferase activity of homogenized organs as indicated, mean & SD. (C) Frozen sections of neonatal lung were analyzed with antibodies and DAPI as indicated. Framed areas are shown in high magnification in Figure 5I. Data from n = 5–8 animals per group from 5 independent experiments. Scale bar: 200 µm.
(TIF)

Figure S7 **CD69 expression of OTI T cells in lung draining lymph nodes of MCMV-infected neonates.** (A) Experimental setup for (B): Neonatal mice were l.p. infected with 5×10^4^ PFU MCMV-2D or MCMV-3D. 4 days after infection 5×10^6^ OTIxCFP T cells were adoptively i.p. transferred. At day 5 lung draining lymph nodes CFP^+^CD8b^+^TCR-Vα2^+^ T cells were analysed for expression of CD69. Representative data from n = 2–4 animals per group.
(TIF)

Figure S8 **Proliferation profile of OTI T cells in lung draining lymph nodes of MCMV-2D-infected neonates.** Neonatal mice were l.p. infected with 5×10^4^ PFU MCMV-2D. 3 days later eFluor 670 labeled GFPxOTI T cells were adoptively transferred and FTY720 was given until analysis. 7 days p.i. lung draining lymph nodes were isolated and GFP^+^ cells analyzed. Representative data from n = 4 animals from 2 independent experiments.
(TIF)

Movie S1 **OTI T cell accumulation in NIFs 1 day post cell transfer.** OTIxGFP T cell (green) accumulation and migration within MCMV-2D versus MCMV-3D-induced neonatal NIFs (infected cells in red) 1 day after cell transfer, second harmonic generation (blue).
(MOV)

Movie S2 **Migration of OTI T cells located peribronchially or within NIFs at 1 day vs. 4 days post cell transfer.** Scene 1 shows peribronchial OTIxGFP T cell (green) migration behaviour and accumulation within a NIF (infected cells in red) of MCMV-3D-infected neonates 1 day after cell transfer. Scene 2 depicts increased OTI T cell (green) motility 4 days after cell transfer in MCMV-3D-infected neonatal lung (infected cells in red), second harmonic generation (blue).
(MOV)

Movie S3 **OTI T cell accumulation in NIFs 2 days post cell transfer.** OTIxGFP T cell (green) accumulation and migration within MCMV-2D versus MCMV-3D-induced neonatal NIFs (infected cells in red) 2 days after cell transfer, second harmonic generation (blue).
(MOV)

Movie S4 **OTI T cell interaction with APCs in NIFs 1 day post cell transfer.** Synapse formation of OTIxCFP T cells (blue) with APCs (yellow) which themselves are in contact with MCMV-3D-infected cells (red) in neonatal NIFs 1 day after cell transfer, second harmonic generation (blue). Surface rendering of same area; arrows point to infected cell and OTI T cell which are connected by APC; infected cell and OTI T cell are briefly highlighted in white.
(MOV)
