# Supplementary figures and images for: Nodular Inflammatory Foci Are Sites of T Cell Priming and Control of Murine Cytomegalovirus Infection in the Neonatal Lung
Source: PLoS Pathog. 2013 Dec 12;9(12):e1003828. doi: 10.1371/journal.ppat.1003828 (PMC3861546; doi:10.1371/journal.ppat.1003828)

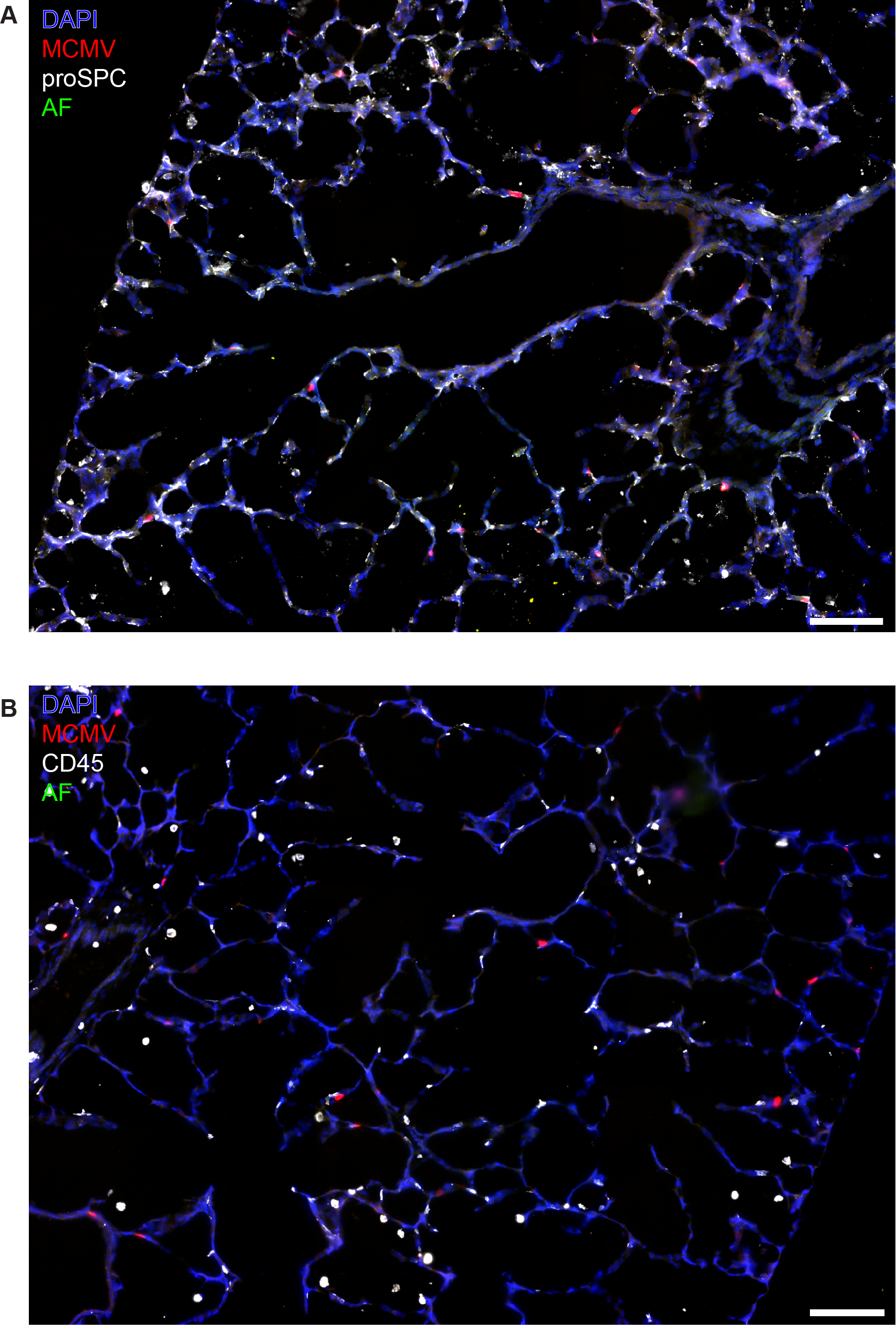

Supplement: Figure S1 — MCMV cell tropism in the neonatal lung at 1 day post infection. (A+B) Neonatal mice were l.p. infected with 5×10^4^ PFU MCMV-3D. One day later mice were sacrificed and lungs explanted. Frozen sections were stained with antibodies and DAPI as indicated. AF, autofluorescence of tissue. Representative from >3 experiments with n = 2–3 animals per experiment. Scale bars: 100 µm (TIF) [file ppat.1003828.s001.tif]

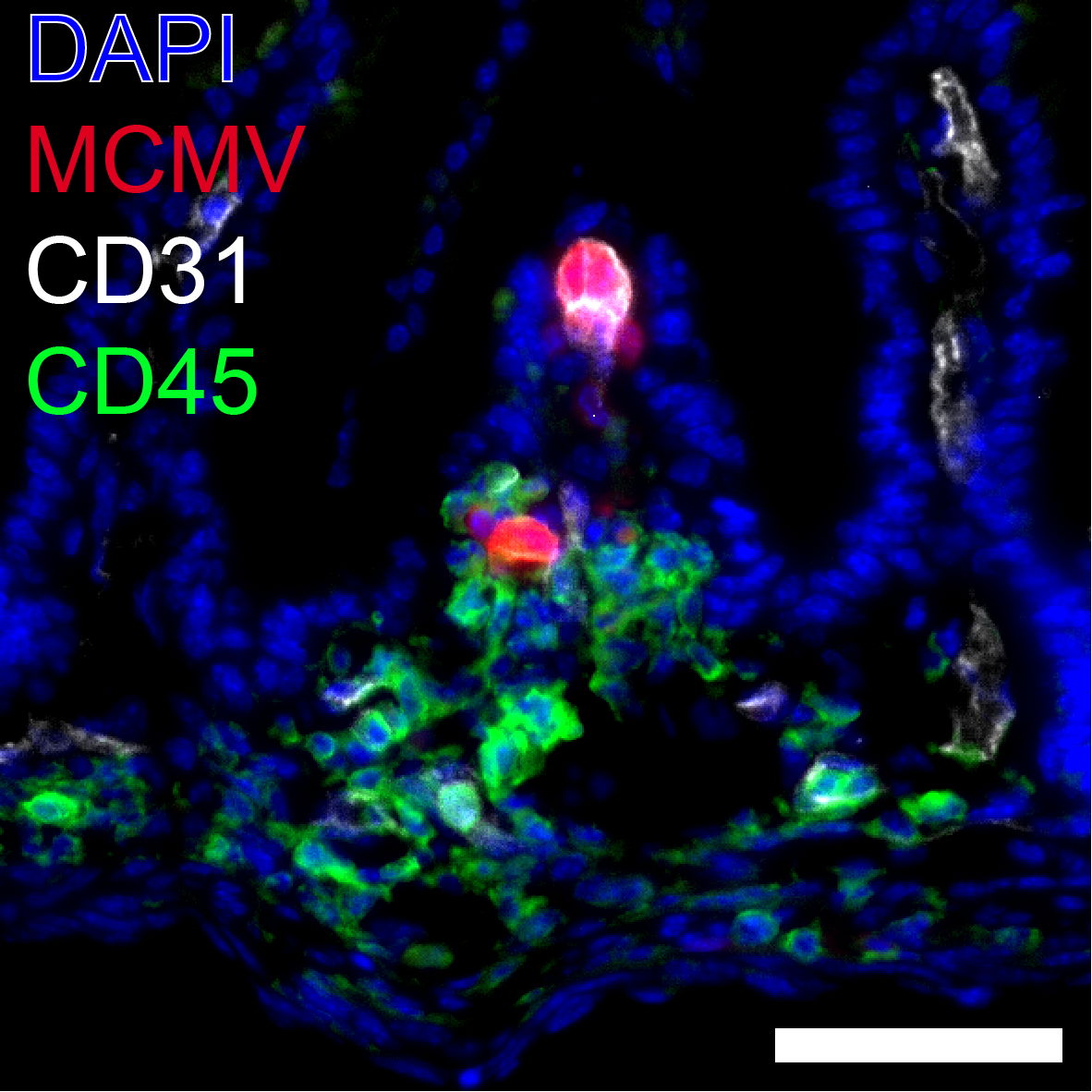

Supplement: Figure S2 — MCMV infection of the neonatal colon. Neonatal mice were l.p. infected with 5×10^4^ PFU MCMV-3D. Intestines were explanted at day 8 after infection. Frozen sections were stained with antibodies and DAPI as indicated. Representative of n = 5 animals from 2 independent experiments. Scale bar: 50 µm. (TIF) [file ppat.1003828.s002.tif]

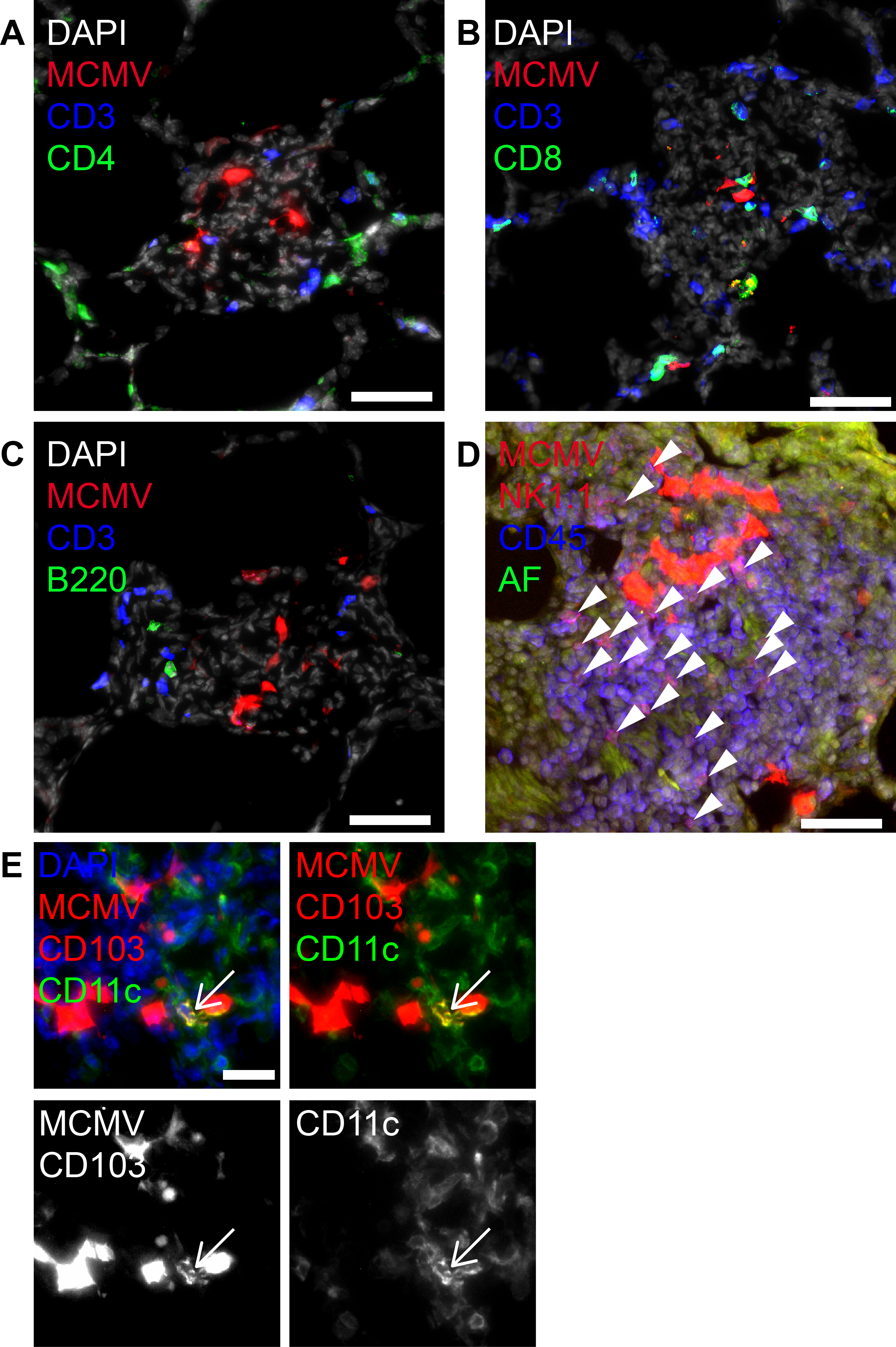

Supplement: Figure S3 — MCMV cell tropism in the neonatal lung at 5 days post infection. (A–E) Neonatal mice were l.p. infected with 5×10^4^ PFU MCMV-3D. 5 days later mice were sacrificed, lungs explanted and frozen sections were stained with antibodies and DAPI as indicated. Arrows point to CD45+NK1.1+ NK cells (D) and CD11c+CD103+ dendritic cell (E). Scale bars: (A–D) 50 µm, (F) 20 µm. (TIF) [file ppat.1003828.s003.tif]

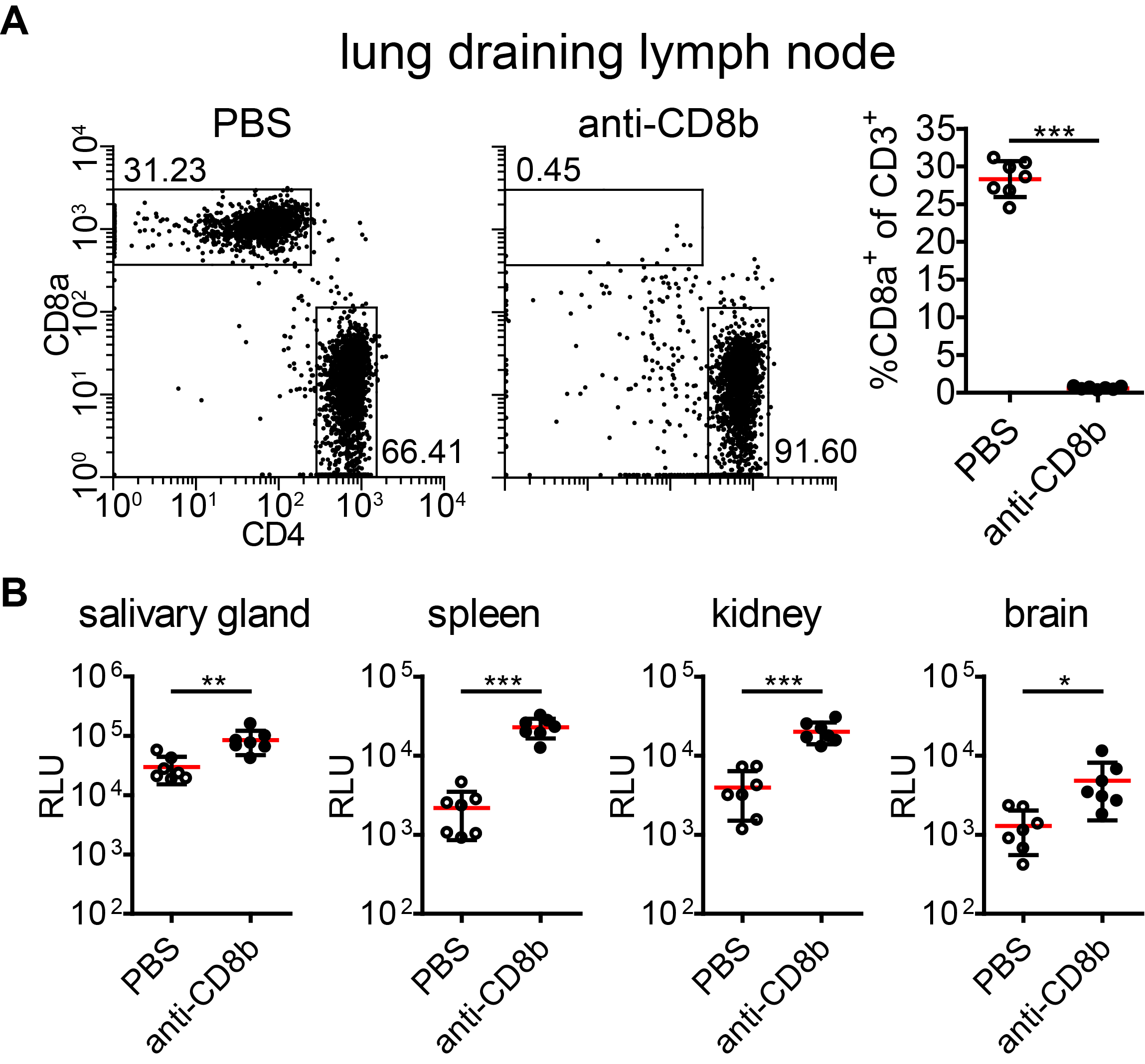

Supplement: Figure S4 — Depletion of CD8+ T cells impairs control of MCMV in neonates. Neonatal mice were l.p. infected with 5×10^4^ PFU MCMV-3D and treated with anti-CD8b antibody or PBS. At day 12 p.i. animals were sacrificed. (A) Lung draining lymph nodes were analysed for the presence of CD8a+ T cells. (B) Luciferase activity was measured from homogenized organs as indicated; mean & SD. Data from n = 7 animals per group from 2 independent experiments. (TIF) [file ppat.1003828.s004.tif]

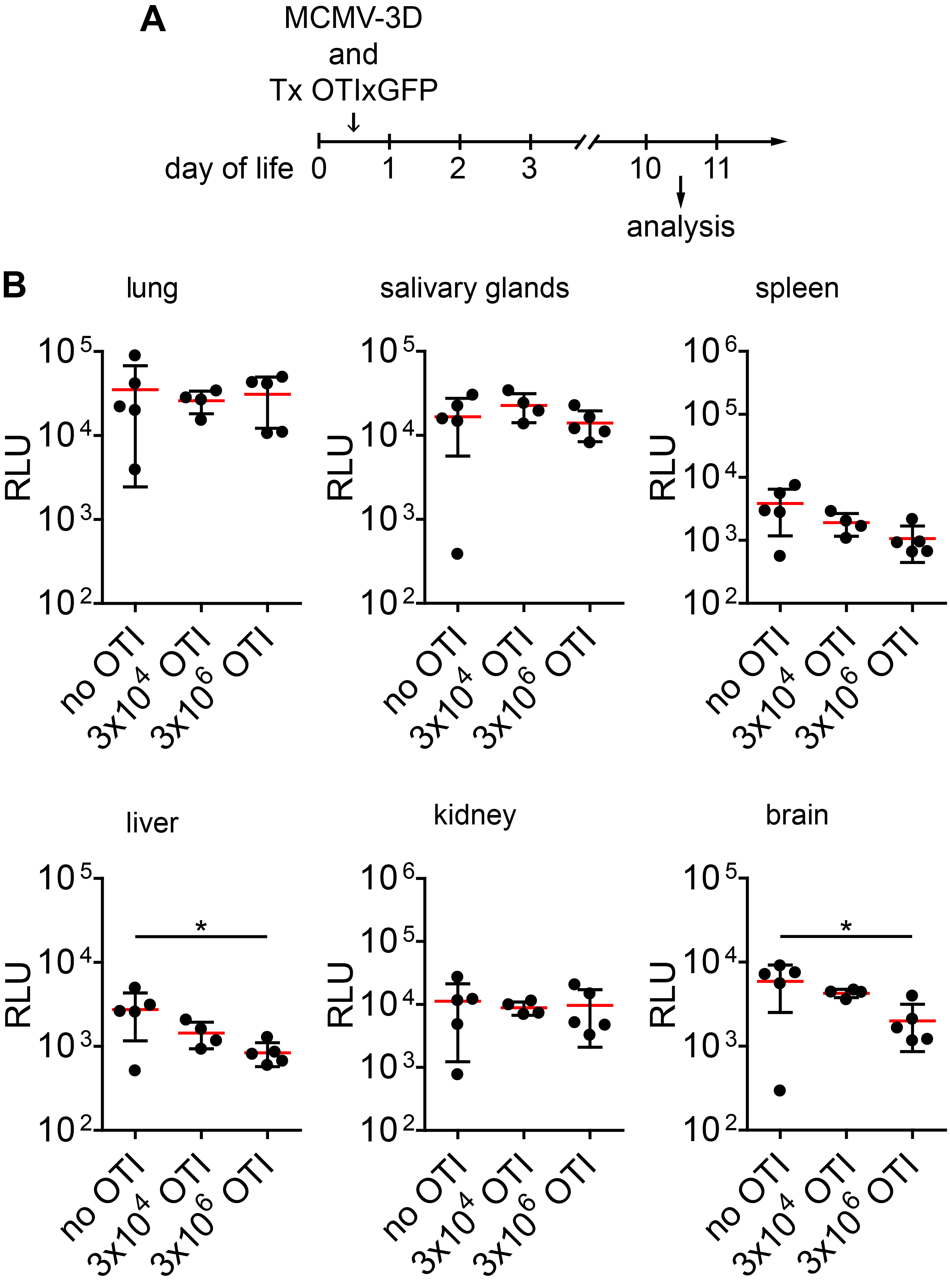

Supplement: Figure S5 — Viral activity 10 days after adoptive transfer of OTI T cell into neonates. (A) Experimental setup for (B): Neonatal mice were l.p. infected with 5×10^4^ PFU MCMV-3D, received indicated numbers of CD8+ OTIxGFP cells intraperitoneally at the same day and were analysed at day 10 p.i. (B) Luciferase activity of homogenized organs as indicated, mean & SD, n = 4–5 animals per group from 2 independent experiments. (TIF) [file ppat.1003828.s005.tif]

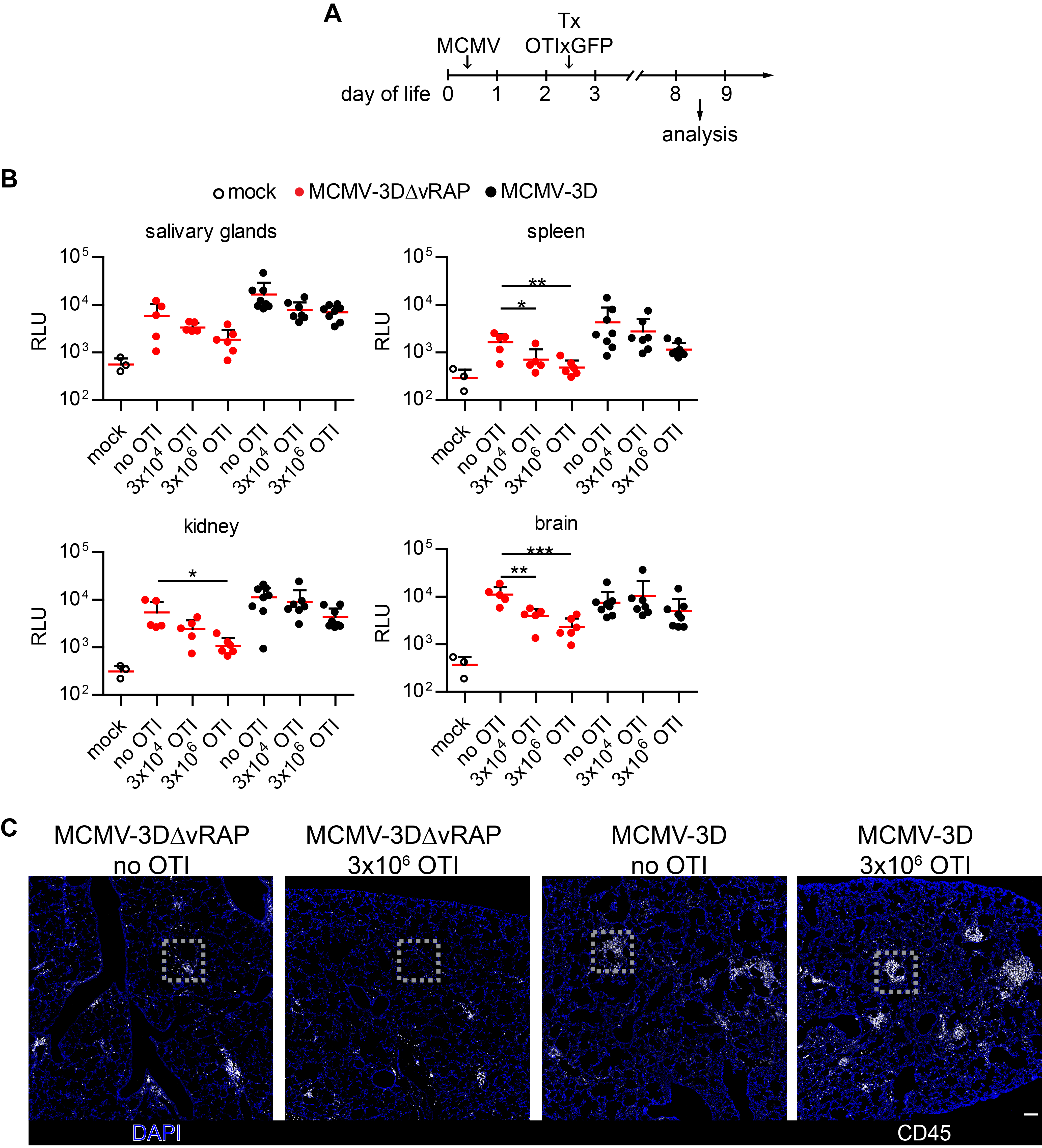

Supplement: Figure S6 — Viral activity 6 days after adoptive transfer of OTI T cell into neonates. (A) Experimental setup for (B+C): Neonatal mice were l.p. infected with 5×10^4^ PFU MCMV-3DΔvRAP or MCMV-3D, received indicated numbers of CD8+ OTIxGFP cells intraperitoneally at day 2 and were analysed at day 8 p.i. (B) Luciferase activity of homogenized organs as indicated, mean & SD. (C) Frozen sections of neonatal lung were analyzed with antibodies and DAPI as indicated. Framed areas are shown in high magnification in Figure 5I. Data from n = 5–8 animals per group from 5 independent experiments. Scale bar: 200 µm. (TIF) [file ppat.1003828.s006.tif]

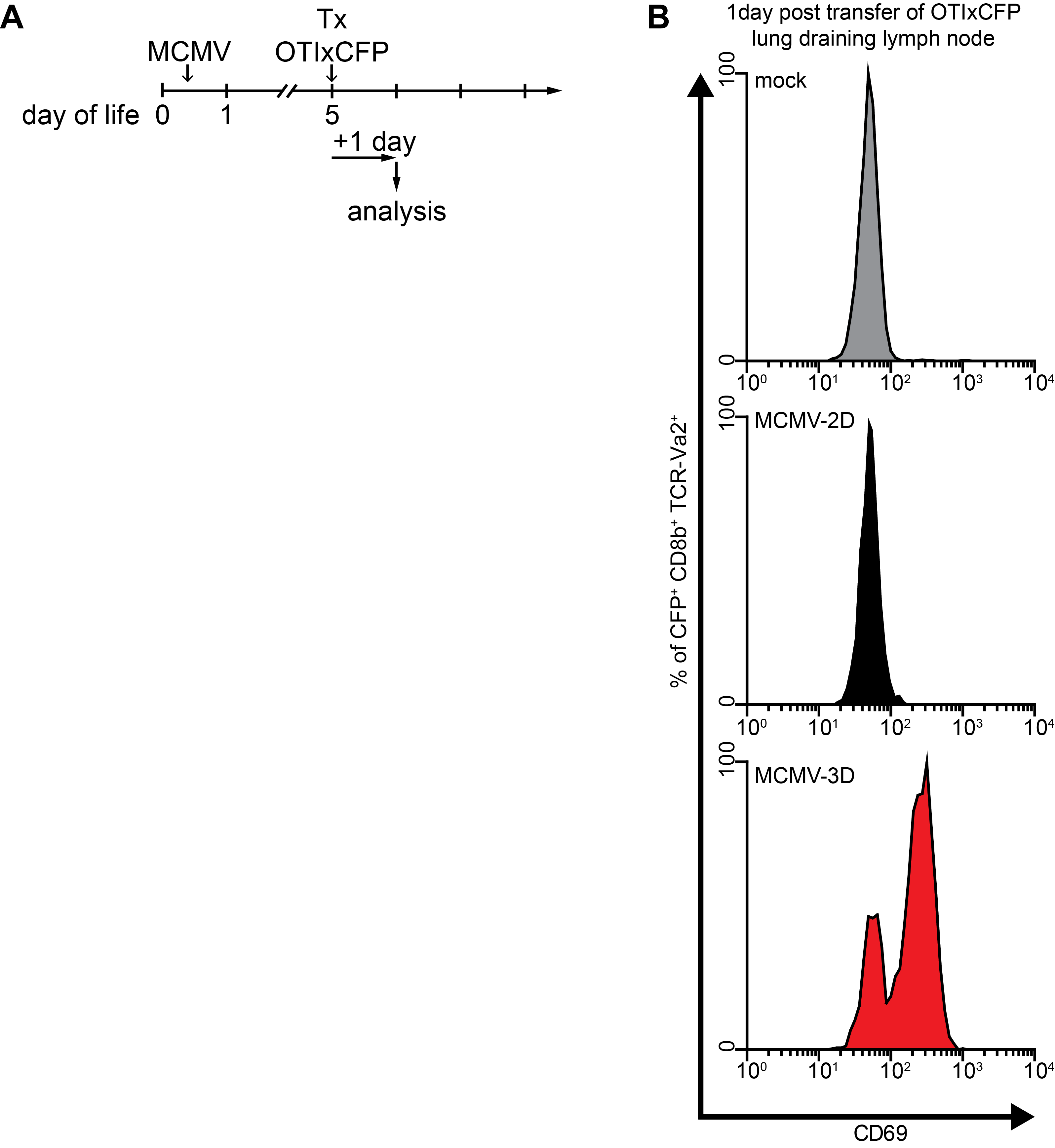

Supplement: Figure S7 — CD69 expression of OTI T cells in lung draining lymph nodes of MCMV-infected neonates. (A) Experimental setup for (B): Neonatal mice were l.p. infected with 5×10^4^ PFU MCMV-2D or MCMV-3D. 4 days after infection 5×10^6^ OTIxCFP T cells were adoptively i.p. transferred. At day 5 lung draining lymph nodes CFP+CD8b+TCR-Vα2+ T cells were analysed for expression of CD69. Representative data from n = 2–4 animals per group. (TIF) [file ppat.1003828.s007.tif]

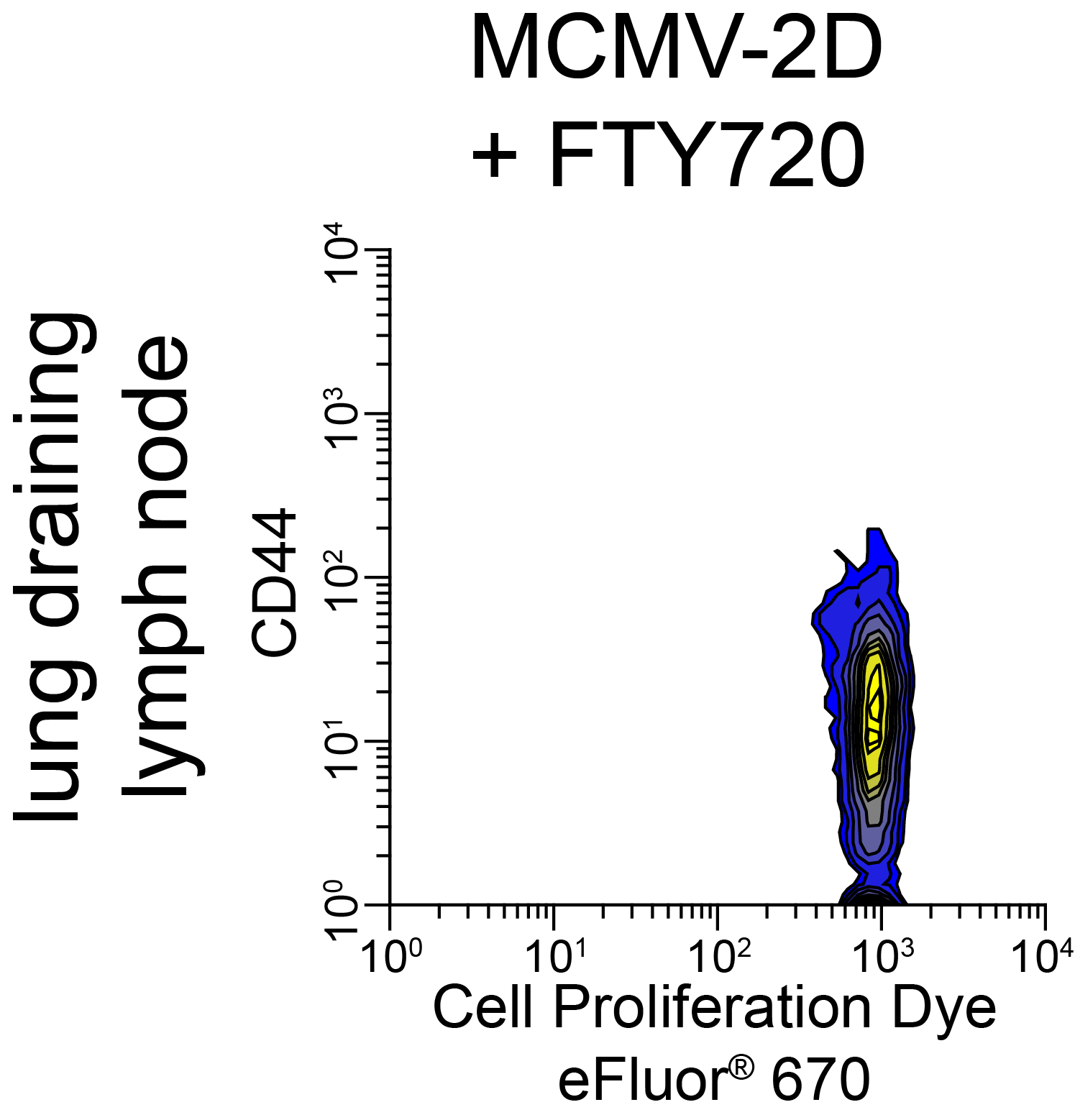

Supplement: Figure S8 — Proliferation profile of OTI T cells in lung draining lymph nodes of MCMV-2D-infected neonates. Neonatal mice were l.p. infected with 5×10^4^ PFU MCMV-2D. 3 days later eFluor 670 labeled GFPxOTI T cells were adoptively transferred and FTY720 was given until analysis. 7 days p.i. lung draining lymph nodes were isolated and GFP+ cells analyzed. Representative data from n = 4 animals from 2 independent experiments. (TIF) [file ppat.1003828.s008.tif]
